# mTORC1-independent TFEB activation via Akt inhibition promotes cellular clearance in neurodegenerative storage diseases

**DOI:** 10.1038/ncomms14338

**Published:** 2017-02-06

**Authors:** Michela Palmieri, Rituraj Pal, Hemanth R. Nelvagal, Parisa Lotfi, Gary R. Stinnett, Michelle L. Seymour, Arindam Chaudhury, Lakshya Bajaj, Vitaliy V. Bondar, Laura Bremner, Usama Saleem, Dennis Y. Tse, Deepthi Sanagasetti, Samuel M. Wu, Joel R. Neilson, Fred A. Pereira, Robia G. Pautler, George G. Rodney, Jonathan D. Cooper, Marco Sardiello

**Affiliations:** 1 Department of Molecular and Human Genetics, Baylor College of Medicine, Jan and Dan Duncan Neurological Research Institute, Texas Children’s Hospital, Houston, Texas 77030, USA; 2 Department of Molecular Physiology and Biophysics, Baylor College of Medicine, Houston, Texas 77030, USA; 3 Department of Basic and Clinical Neuroscience, Maurice Wohl Clinical Neuroscience Institute, Institute of Psychiatry, Psychology & Neuroscience, King's College London, London SE5 9RT, UK; 4 Huffington Center on Aging and Department of Molecular and Cellular Biology, Baylor College of Medicine, Houston, Texas 77030, USA; 5 Department of Molecular Physiology and Biophysics, Dan L. Duncan Cancer Center, Baylor College of Medicine, Houston, Texas 77030, USA; 6 Cullen Eye Institute, Department of Ophthalmology, Baylor College of Medicine, Houston, Texas 77030, USA; 7 School of Optometry, The Hong Kong Polytechnic University, Kowloon, Hong Kong; 8 Cardiovascular Research Institute, Baylor College of Medicine, Houston, Texas 77030, USA

## Abstract

Neurodegenerative diseases characterized by aberrant accumulation of undigested cellular components represent unmet medical conditions for which the identification of actionable targets is urgently needed. Here we identify a pharmacologically actionable pathway that controls cellular clearance via Akt modulation of transcription factor EB (TFEB), a master regulator of lysosomal pathways. We show that Akt phosphorylates TFEB at Ser467 and represses TFEB nuclear translocation independently of mechanistic target of rapamycin complex 1 (mTORC1), a known TFEB inhibitor. The autophagy enhancer trehalose activates TFEB by diminishing Akt activity. Administration of trehalose to a mouse model of Batten disease, a prototypical neurodegenerative disease presenting with intralysosomal storage, enhances clearance of proteolipid aggregates, reduces neuropathology and prolongs survival of diseased mice. Pharmacological inhibition of Akt promotes cellular clearance in cells from patients with a variety of lysosomal diseases, thus suggesting broad applicability of this approach. These findings open new perspectives for the clinical translation of TFEB-mediated enhancement of cellular clearance in neurodegenerative storage diseases.

## Abstract (web summary)

The transcription factor EB (TFEB) is a master regulator of lysosomal biogenesis. Here authors show that trehalose, an mTOR-independent autophagy inducer, alleviates the pathological phenotypes in a mouse model of neurodegenerative disease. Trehalose acts by inhibiting Akt, which normally suppresses TFEB via an mTORC1-independent mechanism.

Neurodegenerative diseases pose a major burden on public health that is expected to increase in the next decades due to the extension of life expectancy and global population aging. Unlike other human health conditions, neurodegenerative diseases have proven to be extraordinarily refractory to attempts to halt or slow their progression. Indeed, no approved treatments exist for any neurodegenerative disease that significantly extend life span or modify clinical progression^1^. Therefore, neurodegenerative diseases represent unmet medical conditions for which the identification of effective, pharmacologically actionable targets is urgently needed.

Mounting genetic and experimental evidence converges on cellular clearance pathways as the main processes implicated in the pathogenesis of neurodegenerative diseases. Indeed, the vast majority of patients with a neurodegenerative condition have aberrant neuronal accumulation of undigested macromolecules, as a result of an overwhelmed or impaired cellular degradative system^2^,^3^. Among the identified causes is the abnormal generation of aggregation-prone proteins, which are less efficiently disposed of by the cell, and genetic defects that directly or indirectly affect the autophagic–lysosomal degradative pathway^4^. Hence, a general paradigm is emerging, which proposes that enhancement of cellular clearance in these disease conditions will help maintain cellular homoeostasis and prevent neuronal cell death^5^,^6^. Our recent identification of a genetic program that oversees lysosomal biogenesis and function has provided a suitable target to manipulate lysosomal degradative pathways^7^. The basic helix-loop-helix transcription factor EB (TFEB) indeed acts as a master regulator of cellular clearance through the enhancement of several processes that include lysosomal proliferation^8^, expression of degradative enzymes^8^,^9^, autophagy^10^, lysosomal exocytosis^11^ and lysosomal proteostasis^12^. *In vivo* studies based on heterologous expression of TFEB have shown improved clearance and amelioration of disease phenotypes in rodent models of neurodegenerative disorders such as Alzheimer’s disease^13^,^14^, tauopathy^15^, Parkinson’s disease^16^ and Huntington’s disease^8^,^17^. An opportunity for pharmacological activation of TFEB has stemmed from cell-based studies that indicate that TFEB is negatively regulated by the mechanistic target of rapamycin complex 1 (mTORC1)^18^,^19^,^20^, the main known factor restricting autophagy induction. Catalytic inhibition of mTORC1 in cells leads to TFEB activation; however, rapamycin—the mTORC1 allosteric inhibitor that along with its analogues is leading research in mTOR-related translational applications—is quite ineffective at activating TFEB^18^,^19^,^20^. Indeed, no pharmacological therapy of TFEB activation has been proposed yet. The identification of alternative routes to activate TFEB is therefore needed to move the field forward in translational applications.

Here we identify the serine/threonine kinase Akt (protein kinase B) as a pharmacologically actionable target that controls TFEB activity independently of mTORC1. We find that the non-reducing disaccharide of glucose, α-D-glucopyranosyl α-D-glucopyranoside or trehalose, an mTOR-independent autophagy inducer^21^, promotes nuclear translocation of TFEB by inhibiting Akt. We show that trehalose administration reduces disease burden in a mouse model of a prototypical neurodegenerative disease that presents with abnormal intralysosomal accumulation of undegraded proteinaceous material. We demonstrate that TFEB activity is modulated by Akt phosphorylation at Ser467, and that Akt pharmacological inhibition promotes cellular clearance in a variety of models of genetic diseases presenting with impairment of lysosomal pathways. Modulation of Akt activity is the subject of intense clinical studies. Therefore, the finding that Akt controls TFEB-mediated clearance opens novel perspectives for future pharmacological therapies of neurodegenerative storage disorders.

## Results

### Trehalose attenuates neuropathology in a model of JNCL

The most documented example of mTORC1-independent activation of cellular clearance is that exerted by trehalose^22^,^23^,^24^,^25^,^26^. We hypothesized that trehalose activates TFEB through a hitherto uncharacterized pathway, and set out to test this hypothesis using a prototypical model of aberrant intralysosomal storage represented by juvenile neuronal ceroid lipofuscinosis (JNCL or Batten disease; OMIM #204200), the most prevalent neurodegenerative disorder of childhood. JNCL is caused by mutations in *CLN3*, a gene involved in the regulation of lysosomal homoeostasis^27^,^28^,^29^, and is characterized by autophagic impairment and intralysosomal accumulation of ceroid lipopigment, which is detectable by confocal and electron microscopy^30^,^31^.

**Figure 1:**
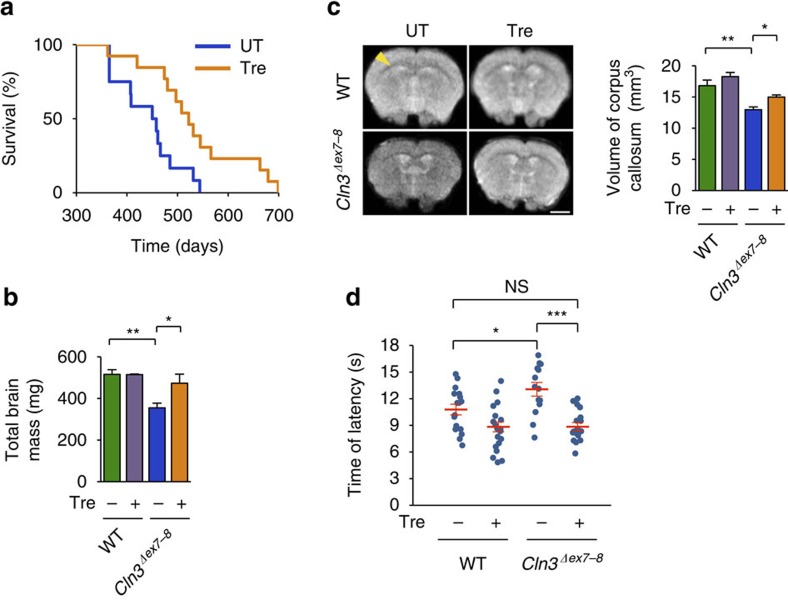
Amelioration of disease pathology in JNCL mice fed with trehalose. (**a**) Trehalose significantly extended survival of *Cln3*^*Δex7–8*^ mice. Treated (Tre) *Cln3*^*Δex7-8*^ mice: *n*=13. Untreated (UT) *Cln3*^*Δex7-8*^ mice: *n*=12. (**b**) Weight of brains from 12-month-old WT and *Cln3*^*Δex7-8*^ mice with or without trehalose treatment. All groups of mice, *n*=4 or 5. (**c**) Fractional anisotropy of brains from 12-month-old WT and *Cln3*^*Δex7-8*^ mice with or without trehalose treatment. Left panel: representative coronal images of the four groups of brains; corpus callosa are indicated by the yellow arrowhead. Right panel: quantification of callosal volume. All groups of mice, *n*=3 or 4. Scale bar, 2 μm. Three-dimensional reconstructions of the corpus callosum in mice from treated and control groups are reported in Supplementary Movies 1–4. (**d**) In the hot plate test, *Cln3*^*Δex7-8*^ mice respond slower when placed on a 50 °C heated metal surface compared with wild-type (WT) littermates, indicating reduced pain sensitivity. Trehalose (Tre) treatment rescued this phenotype in *Cln3*^*Δex7-8*^ mice. All groups of mice, *n*=14–19. Data represent means±s.e.m. *P<0.05, **P<0.01, ***P<0.001.

Oral trehalose administration to C*ln3*^*Δex7-8*^ mice, an established model of JNCL^32^, significantly extended their life span. The median survival of C*ln3*^*Δex7-8*^ mice increased from 454 to 522 days (15% increase, log-rank *P*=0.00566) and the maximum life span increased from 544 to 699 days (28% increase; Fig. 1a). Post mortem examination and neuroimaging studies of JNCL patients have shown generalized brain atrophy, including significant thinning of the corpus callosum (CC) and brainstem^33^,^34^. Magnetic resonance imaging (MRI) studies in JNCL mice reported that CLN3 protein deficiency results in a similar generalized atrophy of the brain, thereby mirroring the human condition^35^. We measured the wet brain weight of 12-month-old *Cln3*^*Δex7-8*^ mice (0.355±0.024 g) and found that it was indeed significantly lower than that of age-matched wild-type (WT) mice (0.516±0.021 g; *P*=0.0016); however, this difference was largely rescued by trehalose treatment (0.473±0.028 g; difference with untreated *Cln3*^*Δex7-8*^ mice, *P*=0.032; Fig. 1b). In contrast, trehalose administration did not affect the body weight of *Cln3*^*Δex7-8*^ or WT mice (Supplementary Fig. 1). We next evaluated the CC volume of fixed brains by MRI analysis. Quantitative measurement of 48 stacks per sample showed that *Cln3*^*Δex7-8*^ mice had a marked reduction in the volume of the CC (12.96±0.43 mm^3^; Supplementary Movie 1) compared with their WT counterparts (16.81±0.89 mm^3^; *P*=0.0081; Fig. 1c; Supplementary Movie 2), which was also rescued by the treatment (15.02±0.33 mm^3^; difference with untreated *Cln3*^*Δex7-8*^ mice, *P*=0.027; Fig. 1c; Supplementary Movie 3). The analysis of WT mice treated with trehalose did not show any significant changes in CC volume (18.27±0.66 mm^3^; Fig. 1c; Supplementary Movie 4).

Six-month-old *Cln3*^*Δex7-8*^ mice exhibited reduced pain sensitivity in a hot plate assay, which was fully restored by trehalose (Fig. 1d). Auditory brainstem response (ABR) analysis in 10-month-old mice showed that *Cln3*^*Δex7-8*^ mice have elevations in ABR thresholds relative to WT mice (*P*=0.01027), indicating low-frequency hearing loss (Supplementary Fig. 2). Trehalose treatment resulted in lower ABR thresholds in both genotypes compared with untreated age-matched controls, indicating protection of auditory function (Supplementary Fig. 2). We also attempted to evaluate retinal function by performing electroretinogram analysis of 11-month-old mice; however, several untreated WT mice showed poor response to the test, indicating severe vision loss. The genetic background of our mouse colony (CD-1) had been previously associated with inherited retinal degeneration in ∼60% of males and other phenotypes decreasing vision^36^,^37^. Thus, an evaluation of treatment-associated changes in electroretinogram could not be performed.

**Figure 2:**
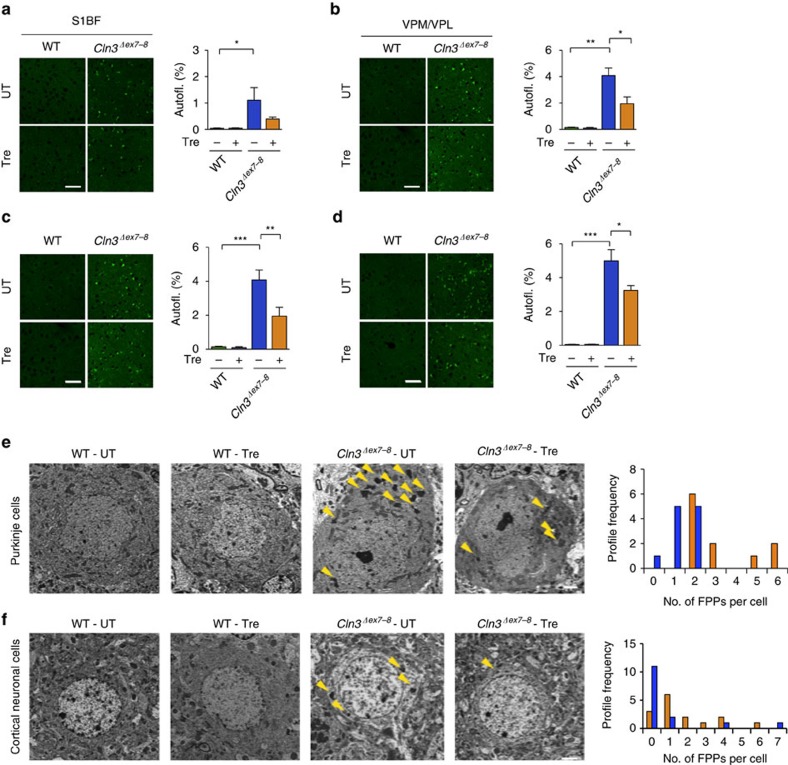
Assessment of storage burden. (**a**,**b**) Confocal images and quantification of the storage material in trehalose-treated (Tre) and untreated mice in the primary somatosensory cortex (S1BF; **a**), and in the interconnected thalamic relay nucleus (VPM/VPL; **b**) at 7 month of age. Thresholding image analysis revealed higher levels of autofluorescent storage material in the cortex and thalamus of *Cln3*^*Δex7-8*^ mice, which is reduced by trehalose treatment. Scale bar, 50 μm. All groups of mice, *n*=3 or 4. (**c**,**d**) Confocal images and quantification of the amount of storage material in 12-month-old trehalose-treated and control mice in the primary somatosensory cortex (S1BF; **c**) and in the interconnected thalamic relay nucleus (VPM/VPL; **d**). Thresholding image analysis revealed higher levels of autofluorescent storage material in the cortex and thalamus of *Cln3*^*Δex7-8*^ mice, which is partially rescued by trehalose treatment. All groups of mice, *n*=3 or 4. Scale bar, 50 μm (**a**–**d**). Data represent means±s.e.m. *P<0.05, **P<0.01, ***P<0.001. (**e**,**f**) TEM analysis of untreated (UT) *Cln3*^*Δex7-8*^ mouse brains show marked accumulation of electron-dense cytoplasmic material (yellow arrowheads) in both Purkinje cells (**e**) and cortical neurons (**f**). Frequency distribution of FPPs counting revealed a significant reduction of FPPs in trehalose (Tre)-treated mice. *n* of cells per group of mice=18. Kolmogorov–Smirnov test was applied for frequency analysis. Scale bars, 2 μm.

**Figure 3:**
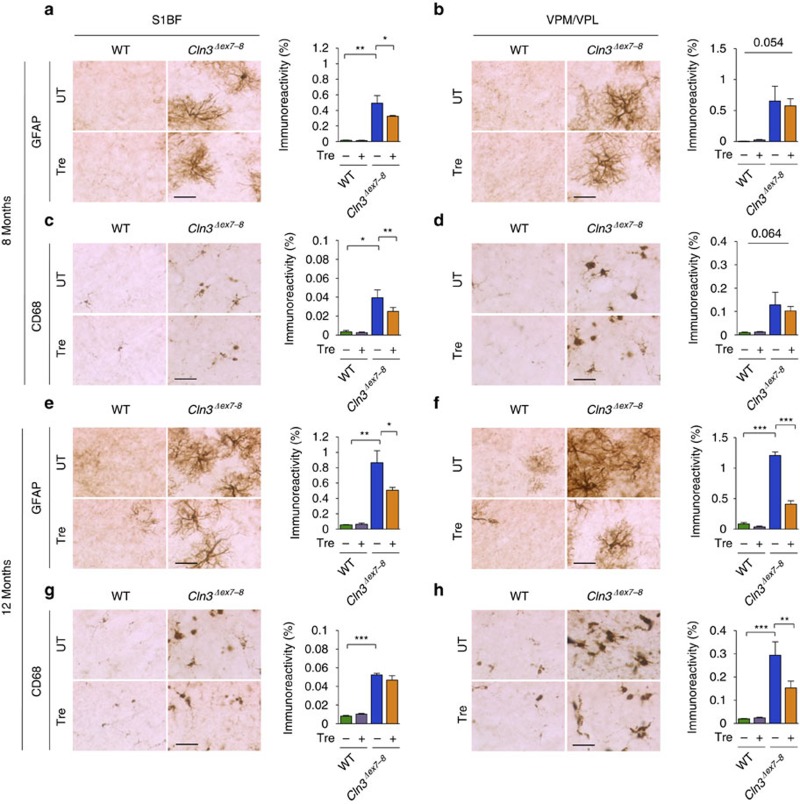
Assessment of neuroinflammation. (**a**,**b**) Analysis and quantification of astrocytosis in trehalose-treated (Tre) and untreated (UT) WT and *Cln3*^*Δex7-8*^ mice at 7 months of age using immunohistochemical staining for GFAP in the primary somatosensory cortex (S1BF; **a**) and in the interconnected thalamic relay nucleus (VPM/VPL; **b**). (**c**,**d**) Analysis and quantification of microglial activation using immunohistochemical staining for CD68 in the S1BF (**c**) and VPM/VPL (**d**) brain regions. Microglial activation is evident in both S1BF and VPM/VPL region of *Cln3*^*Δex7-8*^ mice, which is significantly rescued by trehalose treatment in the S1BF region. All groups of mice, *n*=4 or 5. (**e**,**f**) Analysis and quantification of astrocytosis in trehalose-treated (Tre) and control (UT) mice at 12 months of age using immunohistochemical staining for GFAP in the S1BF (**e**) and in the VPM/VPL (**f**). Trehalose treatment decreased GFAP immunoreactivity in *Cln3*^*Δex7-8*^ mice by 43% in the S1BF region and by 67% in the VPM/VPL region. (**g**,**h**) Analysis and quantification of microglial activation using immunohistochemical staining for CD68, in the S1BF (**g**) and VPM/VPL (**h**) brain regions. Microglial activation is evident in both S1BF and VPM/VPL region of *Cln3*^*Δex7-8*^ mice, which is reduced by 48% in the VPM/VPL region by trehalose treatment. All groups of mice, *n*=3 or 4. Scale bars, 50 μm. Data represent means±s.e.m. *P<0.05, **P<0.01, ***P<0.001.

We next performed microscopic analysis of the brains of *Cln3*^*Δex7-8*^ mice to ascertain whether trehalose modifies the accumulation of ceroid lipopigments. We focused on the primary somatosensory barrel field cortex (S1BF) and on the thalamic ventral posterior medial and lateral nuclei (VPM/VPL), which relays sensory information to the S1BF, because—differently from other regions of the brain—both structures are consistently and severely affected in mouse models of Batten disease^38^. Both regions from 7- and 12-month-old *Cln3*^*Δex7-8*^mice displayed a strong presence of punctate autofluorescent material compared to WT mice, which was found to be significantly reduced by trehalose treatment at both time points (Fig. 2a–d). Transmission electron microscopy (TEM) analysis of *Cln3*^*Δex7-8*^ mouse brains confirmed marked accumulation of electron-dense cytoplasmic material in both Purkinje cells and cortical neurons (Fig. 2e,f). Higher magnification revealed that such electron-dense material consists of the characteristic fingerprint profile structures (Supplementary Fig. 3) previously associated with both human and mouse JNCL pathology^31^,^32^. Trehalose treatment significantly reduced the number of fingerprint profiles in Purkinje cells (*P*=0.047) and cortical neurons (*P*=0.017; Fig. 2e,f), confirming enhancement of cellular clearance in neurons. We next evaluated the effect of trehalose on inflammation. Previous studies reported reactive gliosis and microglial activation in VPM/VPL and S1BF regions of *Cln3*^*Δex7-8*^ mice^38^. Stereological analyses showed that 7-month-old *Cln3*^*Δex7-8*^mice had a marked increase in GFAP and CD68 immunoreactivity in these brain regions compared to age-matched WT mice, thus confirming reactive gliosis and microglial activation (Fig. 3a–d). Both astrocytosis and activation of microglia were exacerbated at 12 months of age (Fig. 3e–h). This progressive neuroinflammation was mitigated by trehalose administration (Fig. 3a,c,e,f,h). Taken together, our data show that treatment with an mTORC1-independent enhancer of clearance reduces brain atrophy, accumulation of lipopigments, and neuroinflammation in a model of a prototypical storage disorder caused by primary impairment of the lysosomal system.

### Trehalose activates TFEB independently of mTORC1

**Figure 4:**
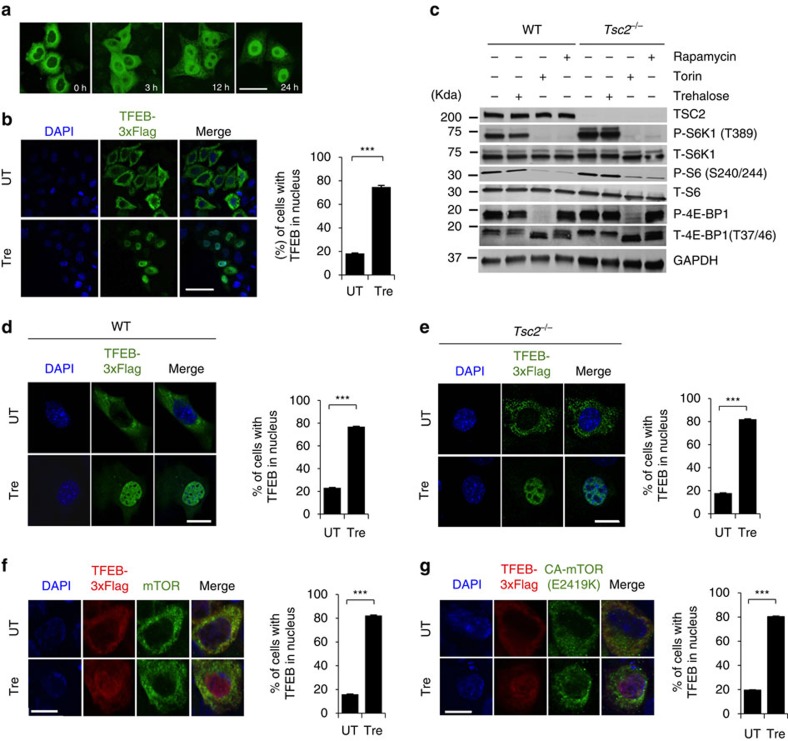
mTORC1-independent nuclear translocation of TFEB on trehalose treatment. (**a**) Confocal microscopy analysis of HeLa/TFEB cells showing time-dependent nuclear translocation of TFEB (green signal) on trehalose treatment. (**b**) Quantification of TFEB subcellular localization (C, cytoplasmic; N, nuclear) after 24 h of trehalose treatment (Tre) or in untreated cells (UT). Scale bars in **a**,**b** is 40 μm. (**c**) Immunoblot analyses show expression levels of substrates downstream of mTORC1. Wild-type (WT) and *TSC2* null MEF cells were treated with trehalose (Tre; 100 mM) for 24 h or left untreated. As controls, cells were treated with Torin 1 (300 nM) or rapamycin (300 nM) for 2 h before extracting the lysates. Phospho- and total S6K1 (P-S6K1 and T-S6K1), phospho- and total S6 (P-S6 and T-S6) and phospho- and total 4E-BP1 (P-4E-BP1 and T-4E-BP1) were detected as readouts of mTORC1 activity. (**d**) WT and (**e**) *TSC2* null MEF cells were transiently transfected with TFEB-3xFLAG and tested for nuclear translocation of TFEB following trehalose administration. (**f**) HeLa cells co-transfected with TFEB-3xFLAG and mTOR or (**g**) TFEB-3xFLAG and constitutively active mTOR (CA-mTOR, C2419K) constructs were treated with trehalose (100 mM for 24 h) or left untreated before immunofluorescent labelling of TFEB (red) and mTOR (green) with FLAG and mTOR antibodies, respectively. Scale bar, 10 μm (**d**–**g**). Data represent means±s.e.m.

The observed reduction of storage material in *Cln3*^*Δex7-8*^ mice suggests that trehalose enhances lysosomal function. TFEB regulates the expression of lysosomal genes by directly binding to the ‘coordinated lysosomal expression and regulation’ (CLEAR) sites that are present at their promoters^8^. To test whether trehalose induces nuclear translocation of TFEB—a hallmark of TFEB activation—we examined cells stably expressing TFEB-3xFLAG (HeLa/TFEB)^8^. Confocal microscopy showed progressive TFEB nuclear translocation on trehalose administration within 24 h (Fig. 4a). Quantification analysis revealed that, in this time frame, cells with nuclear TFEB increased from 20 to >80% (Fig. 4b). Recent reports have demonstrated that mTORC1 phosphorylates TFEB, thereby promoting TFEB cytosolic retention^18^,^19^,^20^. To mechanistically test whether trehalose activates TFEB through an mTORC1-independent pathway, we used two models of constitutive activation of mTORC1. The first model is represented by cells that are null for the tuberous sclerosis complex 2 gene, *Tsc2* (*Tsc2*^*−/−*^)^39^. TSC2 forms a heterodimeric complex with TSC1 that suppresses mTORC1 activity; loss of either TSC2 or TSC1 therefore leads to constitutive mTORC1 activation^40^. Western blot and confocal microscopy analysis of *Tsc2*^*−/−*^ mouse embryonic fibroblasts and control mouse embryonic fibroblasts showed that, unlike the mTORC1 inhibitors (rapamycin and Torin 1), trehalose does not alter mTORC1 signalling (Fig. 4c; Supplementary Fig. 4) and does not modify phosphorylation of TFEB S211 (an mTORC1 target site)^18^,^19^ (Supplementary Fig. 5), but does induce TFEB nuclear translocation even with an active mTORC1 (Fig. 4d,e). The second model we used was obtained with a construct carrying the E2419K amino-acid substitution in the mTOR kinase domain, which results in a constitutively active mTOR (mTOR^E2419K^)^41^. Confocal microscopic analysis showed that trehalose treatment induces nuclear translocation of TFEB in cells transfected with WT mTOR or mTOR^E2419K^ (Fig. 4f,g). Together, these data indicate that trehalose signalling overrides mTORC1 control of TFEB localization. Short interfering RNA (siRNA)-mediated depletion of the mTORC2-specific component RICTOR did not affect TFEB subcellular localization in the presence or absence of trehalose (Supplementary Fig. 6a,b), in agreement with previous studies showing that mTORC2 does not modulate TFEB nuclear translocation^18^.

**Figure 5:**
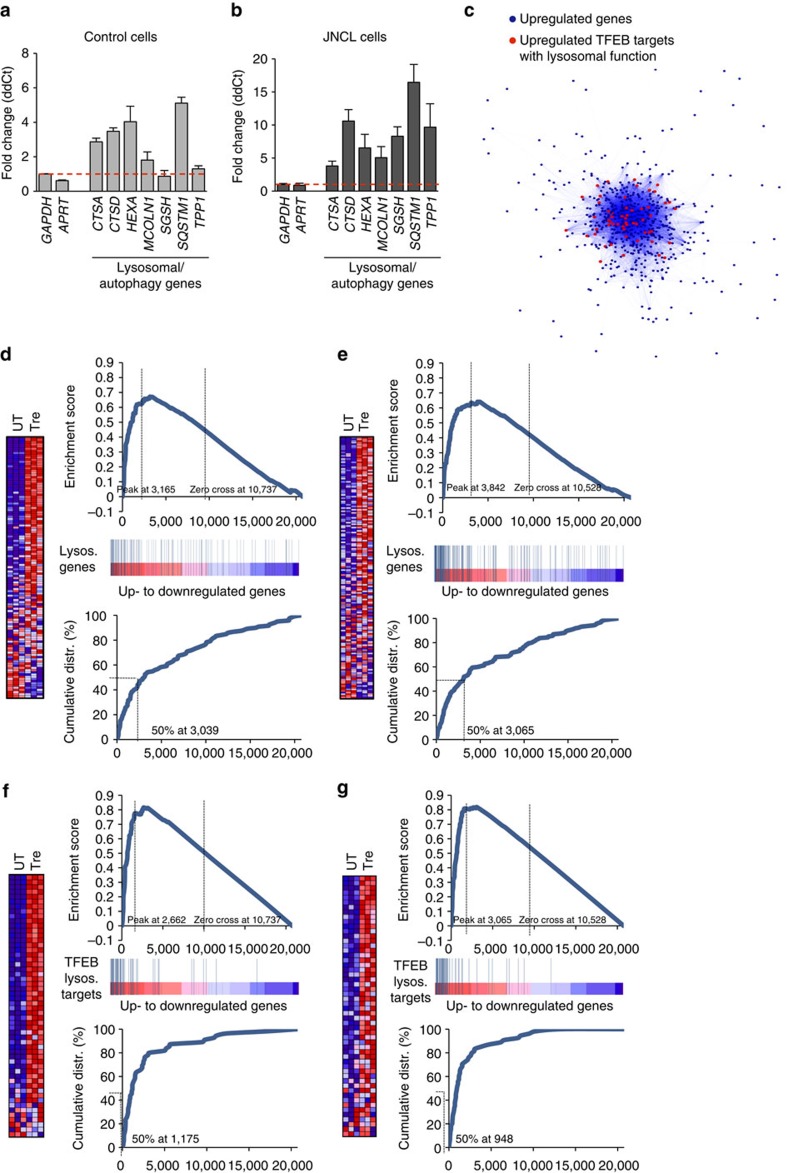
Activation of the CLEAR network by trehalose. (**a**,**b**) Expression analysis of control (CTRL; **a**) and JNCL fibroblasts (**b**) showing upregulation of lysosomal genes on trehalose treatment. Gene expression was normalized relative to the housekeeping gene, *GAPDH*. (**c**) Cytoscape-generated network representing genes upregulated by trehalose administration. Dots (representing genes) are connected by blue lines with colour intensity proportional to the extent of co-regulation. The network has a core of genes with tighter expression relationships containing TFEB lysosomal targets (center of network), while other genes more loosely correlated are found in the periphery of the network. (**d**,**e**) GSEA of transcriptome changes following trehalose administration to CTRL (**d**) and JNCL fibroblasts (**e**), with lysosomal genes. Upper panels show the enrichment plots generated by GSEA of ranked gene expression data (left, red: upregulated; right, blue: downregulated). Vertical blue bars indicate the position of genes in each selected gene set within the ranked lists. Lower panels show the cumulative distribution of lysosomal genes within the ranked lists. The ranking positions that include 50% of analysed genes are indicated. The analysis shows enrichment of lysosomal genes among genes that were upregulated following trehalose administration. (**f**,**g**) GSEA of transcriptome changes following trehalose administration to CTRL (**f**) and JNCL fibroblasts (**g**), with lysosomal genes and TFEB targets with a known role in lysosomal metabolism being reported. TFEB lysosomal targets have a higher ES score than general lysosomal genes, indicating that trehalose preferentially upregulated TFEB targets participating in lysosomal function in both control and JNCL fibroblasts. Data represent means±s.e.m.

We then asked whether trehalose activation of TFEB exerts transcriptional effects that are specific to the CLEAR network, or whether trehalose activates additional programs that might be independent of TFEB. To address this question, we first confirmed that trehalose activates the CLEAR network in human primary cells in normal and pathological conditions. We performed real-time quantitative PCR (qPCR) using messenger RNAs extracted from patient-derived JNCL fibroblasts and control fibroblasts following trehalose administration in culture media. The results showed increased expression of tested CLEAR genes in treated versus untreated fibroblasts with either genetic background (Fig. 5a,b). We next performed microarray expression analysis of JNCL and control fibroblasts following trehalose treatment. Gene ontology analysis of genes with at least a twofold change in expression levels compared with untreated controls showed that the only over-represented class of genes was that related to lysosomal metabolism in both JNCL and control fibroblasts (fold enrichment>5 and *P*<10^−10^ for both analyses). Co-regulation analysis^8^,^9^ revealed that CLEAR genes are at the center of the network of genes induced by trehalose (Fig. 5c), suggesting that TFEB activation may be the first transcriptional response of the cell on trehalose administration. Gene set enrichment analysis (GSEA) of expression changes in control fibroblasts confirmed that the vast majority of lysosomal genes were upregulated on trehalose administration (enrichment score, ES=0.67, *P*<0.0001; Fig. 5d). GSEA of TFEB direct targets with a known role in lysosomal metabolism showed an even greater enrichment (ES=0.82, *P*<0.0001; Fig. 5e), indicating that trehalose specifically activates TFEB-mediated lysosomal regulation. GSEA of gene expression changes in JNCL fibroblasts yielded similar outcomes (Fig. 5f,g); CLN3 deficiency therefore does not disrupt TFEB-mediated lysosomal enhancement.

**Figure 6:**
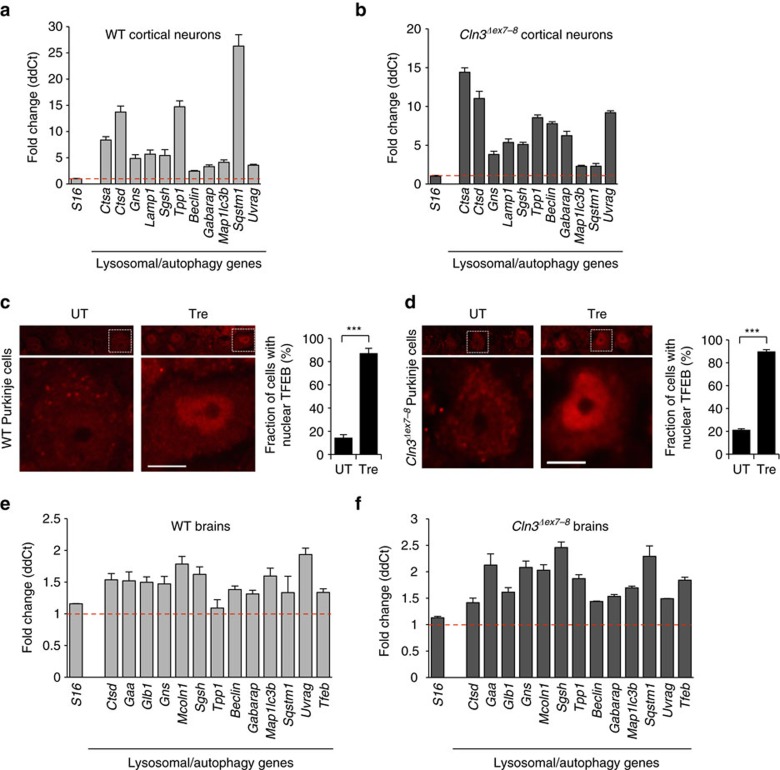
TFEB nuclear translocation and CLEAR network activation *in vivo*. (**a**,**b**) Expression analysis of cultured cortical neurons from WT (**a**) and *Cln3*^*Δex7-8*^ embryos (**b**) at E17.5 shows transcriptional activation of lysosomal genes on trehalose administration. (**c**,**d**) Confocal microscopy of brain sections from WT (**c**) and *Cln3*^*Δex7-8*^ (**d**) mice shows prevalent nuclear distribution of TFEB in Purkinje of treated mice. C and N in bar diagram indicate cytosolic and nuclear distributions, respectively. Scale bar, 20 μm. (**e**,**f**) Expression analysis of brain homogenates from WT (**e**) and *Cln3*^*Δex7-8*^ (**f**) mice on trehalose administration compared to untreated mice, showing transcriptional activation of lysosomal genes. Gene expression was normalized relative to the housekeeping gene, *S16*. The red dashed line indicates relative gene expression in untreated mice. Data represent means±s.e.m.

We confirmed that TFEB activation induces the CLEAR network in primary cortical neuron cultures from WT and *Cln3*^*Δex7-8*^ mice by real-time qPCR (Fig. 6a,b). Immunoblot of proteins extracted from primary cortical astrocyte cultures from WT and *Cln3*^*Δex7-8*^ mice showed increased LAMP1 levels (a marker of lysosomes) on trehalose administration (Supplementary Fig. 7), confirming lysosomal expansion in glial cells. Confocal microscopy of mouse brain sections and expression analysis of whole brain homogenates from WT and *Cln3*^*Δex7-8*^ mice by real-time qPCR showed that oral trehalose administration resulted in TFEB nuclear translocation (Fig. 6c,d) and upregulation of lysosomal and autophagy genes (Fig. 6e,f). TFEB and the CLEAR network are therefore activated *in vivo*.

### Akt controls TFEB activity via phosphorylation at S467

**Figure 7:**
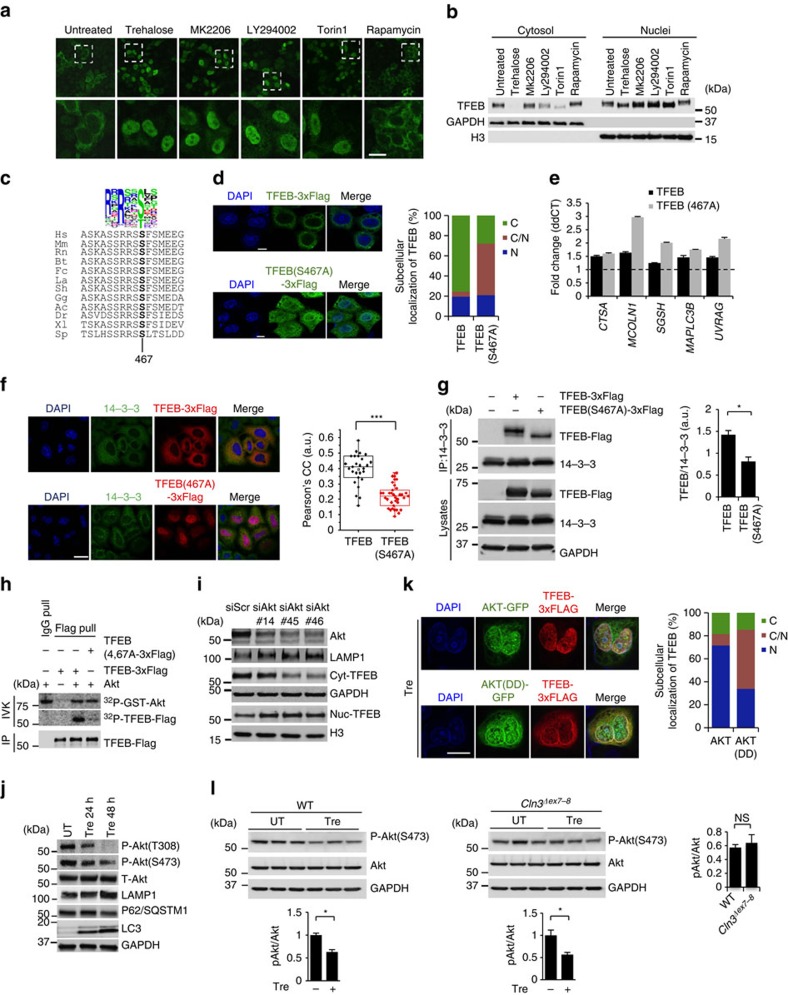
Akt phosphorylates TFEB at Ser467. (**a**) Confocal microscopy analysis of HeLa/TFEB cells showing nuclear translocation of TFEB on addition of trehalose and kinase inhibitors (MK2206 for Akt; LY294002 for PI3K; torin 1 and rapamycin for mTOR). Dashed boxes (upper row) show the location of the higher power inserts (lower row). (**b**) Subcellular fractionation of HeLa/TFEB cells incubated with the same kinase inhibitors. (**c**) Multi-alignment of TFEB amino-acid sequences from the following species: Ac, *Anolis carolensis*; Bt, *Bos taurus*; Dr, *Danio rerio*; Fc, *Felix catus*; Gg, *gallus gallus*; Hs, *Homo sapiens*; La, *Loxodonta africana*; Mm, *Mus musculus*; Rn, *Rattus Norvegicus*; Sh, *Sarcophilus harrisii*; Sp, *Strongylocentrotus purpuratus*; Xl, *Xenopus laevis*. A consensus logo of Akt phosphorylation sites (generated at http://weblogo.berkeley.edu/logo.cgi) is aligned with TFEB sequences. Position 467 refers to the human protein sequence. (**d**) Subcellular localization of TFEB and TFEB(S467A). (**e**) Expression analysis of lysosomal and autophagy genes in HeLa cells transfected with TFEB or TFEB(S467A). Gene expression was normalized relative to the housekeeping gene, *GAPDH*. The dashed line indicates relative gene expression in cells transfected with an empty vector. (**f**) Co-localization assay of 14-3-3 proteins and TFEB-Flag or TFEB(S467A) in HeLa cells. (**g**) Co-immunoprecipitation assays of TFEB or TFEB(S467A) with 14-3-3 proteins. (**h**) Akt *in vitro* kinase assay. Recombinant active AKT1 and purified TFEB-Flag or TFEB(S467A)-Flag were incubated in the presence of [^32^P]ATP, revealing that Akt phosphorylates TFEB and that this reaction requires S467. (**i**) *AKT* silencing mediated by three different *AKT* siRNAs resulted in TFEB nuclear translocation and lysosomal expansion as indicated by western blot analysis. (**j**) Time course analysis of HeLa cells shows trehalose-induced AKT inactivation and increase of autophagic flux as indicated by LAMP1, p62 and LC3 markers. (**k**) HeLa cells co-transfected with TFEB-FLAG and either AKT-GFP or AKT(DD)-GFP were treated for 24 h with trehalose before immunofluorescence labelling of TFEB (red) and AKT-GFP (green). DAPI indicates the nucleus of cells. (**l**) Diminished activation of AKT was observed in WT and *Cln3*^*Δex7-8*^ brain homogenates from trehalose-treated mice. Scale bars, 10 μm (**a**,**e**,**f**,**k**). Data represent means±s.e.m. *P<0.05.

Our data indicate that a pharmacologically actionable pathway activates TFEB and enhances cellular clearance, independent of mTORC1. In the eukaryotic cell, regulatory pathways tend to be based on redundant, dynamically stratified signalling networks that maximize output effectiveness while preserving adaptability to ever-changing cell conditions^42^,^43^. Thus, we reasoned that upstream regulators of TFEB might lie in the same signalling cascade that includes mTORC1. The kinase activity of mTORC1 is tightly regulated by TSC2, which becomes inactive on phosphorylation by the PI3K/Akt signalling pathway^44^. Because inhibition of either PI3K or Akt resulted in TFEB nuclear translocation similar to mTORC1 inhibition by Torin 1 (Fig. 7a,b), we investigated whether Akt directly regulates TFEB activity independent of mTORC1. We used *Tsc2*^*−/−*^ cells to test TFEB responsiveness to Akt activity under conditions of constitutive activation of mTORC1. Consistent with previous studies^44^, Akt activity could be stimulated by serum repletion in *Tsc2*^*−/−*^ cells, where the mTORC1 pathway is insensitive to serum removal or stimulation (Supplementary Fig. 8a). Importantly, serum re-stimulation of serum-starved *Tsc2*^*−/−*^ cells resulted in TFEB nucleus-to-cytosol translocation, which was prevented by preincubation with the Akt inhibitor MK2206 (Supplementary Fig. 8b). Thus, Akt activity is required for TFEB cytosolic localization on serum stimulation independent of mTORC1. We also checked possible interdependence with GSK3β, another factor modulating TFEB subcellular localization^45^,^46^,^47^. An immunoblot analysis showed no detectable effect of trehalose on GSK3β activity (Supplementary Fig. 9a), and confocal microscopic analyses showed that both trehalose and MK2206 were able to induce nuclear translocation of TFEB in cells expressing constitutively active GSK3β (CA-GSK3β/S9A-GSK3β; Supplementary Fig. 9b). In a reciprocal experiment, the GSK3β inhibitor CHIR-99021 promoted nuclear translocation of TFEB in cells expressing constitutively active Akt (Akt^308D/473D^ or Akt-DD)^48^ (Supplementary Fig. 9c). Thus, these results indicate that Akt and GSK3β regulate TFEB independently. We also verified that trehalose does not inhibit the extracellular signal -regulated kinase (ERK), a previously reported modifier of TFEB activity^10^ (Supplementary Fig. 10).

To determine whether Akt directly phosphorylates TFEB, we first built a position weight matrix (PWM) of Akt target sequences by using experimentally validated Akt substrates, and used Akt PWM to scan TFEB amino-acid sequences from multiple species. This analysis identified S467 as a conserved candidate phosphoacceptor motif for Akt (Fig. 7c). A mutant form of TFEB (S467A) shifted to a lower molecular weight when analysed by western blot (Supplementary Fig. 11) and displayed reduced cytosolic localization and increased dual nuclear-cytosolic distribution (Fig. 7d) similar to mutants for mTORC1 target sites (Supplementary Fig. 12). Importantly, TFEB(S467A) showed increased ability to induce the expression of TFEB target genes compared to WT TFEB (Fig. 7e). Cytosolic TFEB has been shown to interact with the 14-3-3 proteins^18^,^19^. As expected, TFEB(S467A) showed diminished co-localization and interaction with the 14-3-3 proteins likely due to its increased nuclear localization (Fig. 7f,g). TFEB(S467A) also displayed nuclear localization in cells with constitutively active mTORC1 (Supplementary Fig. 13).

An *in vitro* Akt kinase assay showed that Akt phosphorylates purified TFEB, but not the S467A mutant form of TFEB (Fig. 7h). Therefore, these results identify TFEB as a direct phosphorylation substrate of Akt and demonstrate that S467 is a key residue for such phosphorylation. Transfection of bicistronic TFEB-Flag–internal ribosomal entry site (IRES)–green fluorescent protein (GFP) and TFEB(S467A)-Flag–IRES–GFP vectors showed that the mutant TFEB protein was more stable than WT TFEB (Supplementary Fig. 14), thus indicating that Akt also regulates TFEB stability. *AKT* knockdown enhanced TFEB nuclear translocation and increased LAMP1 expression (Fig. 7i), thus confirming that Akt negatively regulates TFEB activity. Importantly, trehalose inhibited Akt activity while increasing autophagic flux (Fig. 7j), and expression of constitutively active Akt (Akt-DD) abolished the effect of trehalose on TFEB nuclear translocation (Fig. 7k). These experiments demonstrate mechanistically that Akt inhibition mediates trehalose activation of TFEB. Trehalose-mediated Akt inhibition was confirmed in the brain of trehalose-treated mice (Fig. 7l). Co-immunoprecipitation (IP) experiments confirmed that Akt interacts with TFEB (Supplementary Fig. 15a) and that such interaction does not substantially change when using the S467A mutant version of TFEB (Supplementary Fig. 15b), suggesting that trehalose affects the activity of Akt rather than its interaction with TFEB. We also tested whether TFEB paralogues, MITF and TFE3, are responsive to Akt activity. Confocal microscopic analysis of HeLa cells transfected with MITF and TFE3 constructs showed that inhibition of Akt with MK2206 promoted nuclear translocation of these two factors (Supplementary Fig. 16), thus suggesting possible conservation of this regulatory mechanism.

**Figure 8:**
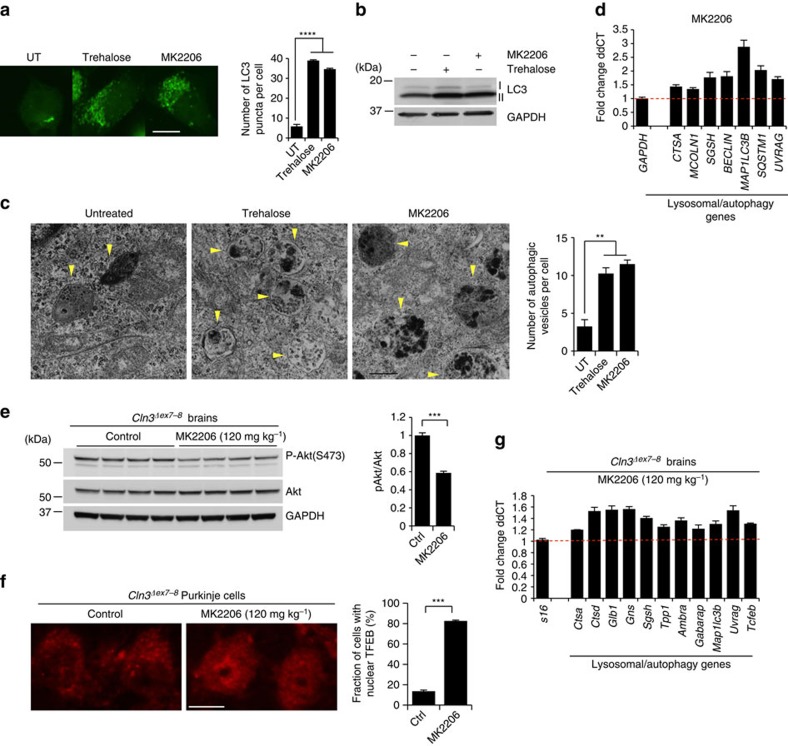
Akt inhibition promotes TFEB nuclear translocation and activation of the CLEAR network. (**a**) LC3 staining showing increased number of puncta in cells treated with trehalose or MK2206. (**b**) Immunoblot analysis of LC3 lipidation. (**c**) Micrographs of HeLa cells showing increased number of autophagic vesicles (yellow arrows) in samples treated with trehalose or MK2206. (**d**) Expression analysis of lysosomal and autophagy genes in HeLa cells treated with MK2206. Gene expression was normalized relative to the housekeeping gene, *GAPDH*. The dashed line indicates relative gene expression in untreated cells. (**e**–**g**) Intraperitoneal injection of MK2206 in *Cln3*^*Δex7-8*^ mice shows inactivation of Akt (**e**), nuclear translocation of TFEB (**f**) and upregulation of lysosomal and autophagy genes (**g**). Scale bar, 10 μm (**a**), 50 nm (**c**) and 20 μm (**f**).

Akt is the subject of intensive clinical investigation due to its involvement in cancer. Among Akt modulators, MK2206 is a potent Akt oral inhibitor that is currently in pre-clinical and phase I clinical studies^49^,^50^. Similar to trehalose, administration of MK2206 to HeLa cells resulted in increased number of LC3 puncta (Fig. 8a), increased LC3-II protein levels (Fig. 8b), and increased number of autophagic vesicles as observed by TEM (Fig. 8c), indicating that MK2206 activates autophagy. In addition, MK2206 treatment also upregulated the expression of lysosomal and autophagy genes (Fig. 8d). Intraperitoneal injection of MK2206 led to inhibition of Akt activity (Fig. 8e) and resulted in TFEB nuclear translocation (Fig. 8f) in the mouse brain, which in turn promoted upregulation of lysosomal and autophagy genes as detected by expression analysis of whole brain homogenates (Fig. 8g). Together, these data provide evidence that pharmacological inhibition of Akt enhances the autophagic-lysosome pathway *in vitro* and *in vivo*.

**Figure 9:**
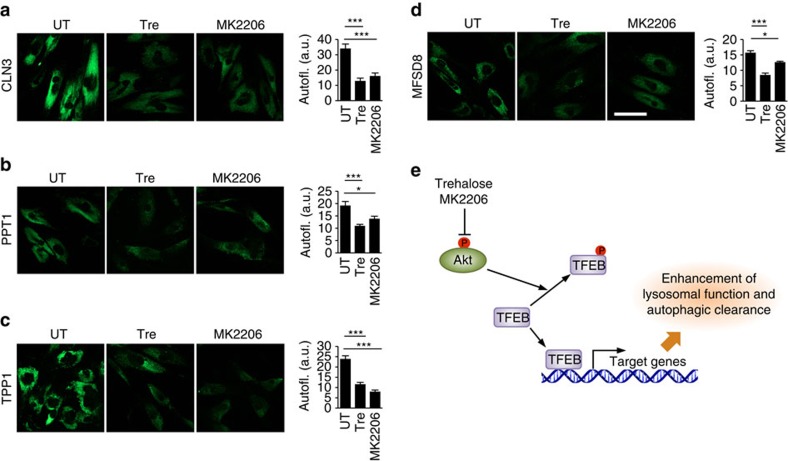
Pharmacological inhibition of Akt enhances cellular clearance in patient-derived cells. (**a**–**d**) Confocal microscopy analysis of primary fibroblasts with defective CLN3 (c.461-677del; **a**), PPT1 (c.665 T>C, p.L222P; **b**), TPP1 (c.380G>A, p.R127Q; g.3556, IVS5-1G>C; **c**) or MFSD8 (c.103C>T, p.R35X; **d**) shows that MK2206 and trehalose induce clearance of ceroid lipopigment deposits (green). Defective proteins are indicated. More than 60 cells have been analysed for each panel. Scale bar, 30 μm. (**e**) Schematic diagram for Akt-dependent trehalose activation of TFEB. Data represent means±s.e.m. *P<0.05, **P<0.01, ***P<0.001.

We finally tested whether MK2206 modulates cellular clearance using accumulation of ceroid lipopigment as a direct readout. Inhibition of Akt by MK2206 in JNCL fibroblasts indeed resulted in clearance of ceroid lipopigment similar to that observed with trehalose treatment (Fig. 9a), which was reversed by withdrawal of MK2206 or trehalose (Supplementary Fig. 17). We then used cell lines with mutations in other lysosomal genes to test whether Akt inhibition enhances cellular clearance independently of the molecular pathways whose dysfunction leads to the buildup of aberrant intralysosomal storage. We first tested a cell line-bearing mutations in the gene encoding palmitoyl-protein thioesterase-1 (PPT1), an enzyme involved in protein degradation whose deficiency results in intralysosomal storage of palmitoylated proteins and neurodegeneration (OMIM #600722). Previous work has shown that chemical cleavage of thioester linkage in palmitoylated proteins results in neuroprotection in a mouse model of PPT1 deficiency, thus directly linking the accumulation of undegraded proteins to disease pathogenesis^51^. Inhibition of Akt using MK2206 dramatically decreased the intralysosomal protein buildup in patient-derived primary fibroblasts bearing mutations in *PPT1* (Fig. 9b). Similarly, MK2206 administration decreased protein buildup in primary fibroblasts with defective tripeptidyl peptidase I (TPP1; Fig. 9c), an exopeptidase that sequentially removes tripeptides from the N termini of proteins and whose deficiency causes neurodegeneration (OMIM #607998). Finally, we tested a model of intralysosomal storage caused by the deficiency of the transmembrane transporter, MFSD8 (OMIM #611124). While the molecular pathway linking MFSD8 function to buildup of proteinaceous material is currently unknown, such aberrant storage is associated with neurodegeneration. Akt inhibition with MK2206 resulted in markedly enhanced cellular clearance in primary fibroblasts defective for MFSD8 (Fig. 9d). Together, these data demonstrate that Akt inhibition can enhance cellular clearance downstream and independent of the primary disrupted pathway. On the basis of on our results, we propose that the Akt-TFEB signalling pathway (schematized in Fig. 9e) may be leveraged with small molecules to improve clearance of toxic material in neurodegenerative diseases.

## Discussion

This study identifies Akt control of TFEB activity as an mTORC1-independent, pharmacologically actionable target with potential relevance for the treatment of neurodegenerative storage diseases. TFEB is indeed a central hub controlling lysosome-based cellular clearance^8^, whose potential therapeutic relevance has been demonstrated in models of the major neurodegenerative diseases, including Alzheimer’s disease, Parkinson’s disease and Huntington’s disease through proof-of-principle studies based on heterologous expression of TFEB^8^,^13^,^14^,^15^,^16^,^17^. Our data using Batten disease mice as an *in vivo* model of neuronal intralysosomal storage demonstrate that lysosomal enhancement can be leveraged to counteract defects in clearance pathways due to primary impairment of lysosomal homoeostasis and function. These findings are relevant for lysosomal storage disorders that, like the juvenile form of Batten disease, are caused by the deficiency of a membrane-bound protein for which approaches based on bone marrow transplantation or gene therapy are inherently difficult to apply^52^. More broadly, our findings are of potential interest for neurodegenerative storage diseases for which validated targets of treatment have still not been established, yet experimental evidence has identified enhancement of cellular clearance pathways as a candidate therapeutic target. Pioneering genetic and mechanistic studies have indeed unveiled strong links between pathogenic mechanisms and lysosomal function in these diseases^2^,^3^,^4^,^5^,^6^.

Understanding how to pharmacologically control TFEB activity is urgently needed to move the field forward and help set-up clinical studies to evaluate the efficacy of TFEB-mediated lysosomal enhancement in neurodegenerative disease. Recent cell-based studies have shown that TFEB activity may be regulated by mTORC1-mediated phosphorylation at specific serine residues in response to changes in the nutritional status^18^,^19^,^20^. This represented a significant discovery because mTORC1 itself is known to be involved in the regulation of autophagy and has therefore been the subject of pre-clinical investigation in models of neurodegenerative diseases. Results from multiple studies indicated that autophagy upregulation via mTORC1 inhibition attenuates neurodegenerative pathology in mouse models of Huntington’s disease^53^, Alzheimer’s disease^54^,^55^, tauopathy^56^, frontotemporal lobar dementia^57^, spinocerebellar ataxia type III (ref. 58) and familial prion disease^59^. mTORC1, however, acts as a central regulatory hub controlling anabolic pathways such as cell growth by modulating the synthesis of proteins, lipids and nucleotides^60^, and long-term mTORC1 inhibition results in induction of immunosuppression and impaired wound healing^3^,^61^. Clinically, mTORC1 inhibition is obtained by using rapamycin, the first identified mTORC1 allosteric inhibitor^62^, or rapamycin analogues that present improved pharmacological profiles. However, allosteric inhibition of mTORC1 by rapamycin has small or no effects on TFEB activation^18^,^19^,^20^. Our identification of an mTORC1-independent route to pharmacologically activate TFEB offers a new avenue to test TFEB-mediated enhancement of cellular clearance in neurodegenerative diseases. Intriguingly, TFEB pharmacological activation and mTORC1 allosteric inhibition could be used as orthogonal, synergic activators of autophagic–lysosomal clearance pathways, ideally identifying drug dosages that would minimize potential side effects of either drug. The increased availability of Akt inhibitors and, importantly, of dual PI3K/mTOR inhibitors may therefore prove beneficial for future pre-clinical and clinical studies.

Akt, a member of the AGC serine/threonine kinase family, plays key roles in the cell survival and apoptosis inhibition. Abnormal activation of Akt may occur through mechanisms such as Akt mutation or dysregulation of upstream signalling pathways, and is an important driver of malignant progression and chemoresistance^63^. This makes Akt a potential therapeutic target for cancer treatments. Intense pre-clinical and clinical effort is indeed being placed on characterizing downstream pathways regulated by Akt and in testing chemical inhibition of Akt in cancer patients^49^,^50^,^64^,^65^. Interestingly, pioneering studies have shown that Akt regulates macroautophagy^66^ and chaperone-mediated autophagy^67^. While it remains to be determined how a disaccharide like trehalose modulates Akt activation, the finding that Akt regulates lysosomal function via TFEB adds a crucial layer in the characterization of Akt’s role in autophagic–lysosomal clearance pathways and offers a novel angle in understanding the cellular processes that are impacted by the clinical use of Akt inhibitors. Since PI3K-Akt pathway plays a key role in the integration of signals from secreted growth factors to stimulate mTORC1 activity, it is also interesting that Akt inhibition by trehalose does not inhibit mTORC1. In response to growth factors, Akt phosphorylates and inhibits the TSC2, which acts as a negative regulator of mTORC1 by maintaining the mTORC1 direct activator, Rheb, in its inactive GDP-bound state^68^. Another upstream regulator of mTORC1 that acts in parallel to Akt via the same TSC2/Rheb cascade is ERK, which integrates signals from growth factors through the Ras-ERK pathway. Similar to Akt, ERK also inhibits TSC2. We found that trehalose inhibits Akt but not ERK activity; therefore, it is possible that active ERK is sufficient to keep TSC2 inactive, thus resulting in an unmodified mTORC1 signalling. TSC proteins also integrate signals from other pathways, thus additional layers of regulation might be responsible for mTORC1 insensitivity to trehalose.

In summary, the identification of Akt as an mTORC1-independent regulator of TFEB opens new perspectives for the pharmacological control of TFEB-mediated cellular clearance. Akt modulation of TFEB might be exploited therapeutically to enhance cellular clearance in neurodegenerative storage disorders, and the availability of drugs that target the Akt-TFEB signalling pathway warrants future studies aimed at the clinical translation of TFEB-mediated lysosomal enhancement in neurodegenerative diseases.

## Methods

### Cell culture and treatment

Control (Coriell Institute, USA) and JNCL fibroblasts (Gaslini Institute, Italy) were grown in DMEM (1:1, HyClone) supplemented with 10% heat-inactivated fetal bovine serum (FBS, Atlanta Biologicals), 2 mM L-glutamine, 100 U ml^−1^ penicillin and 100 mg ml^−1^ streptomycin (Invitrogen). HeLa cells were incubated for 2 h with LY294002 (50 mM, Cell Signaling), Torin 1 (300 nM, Cayman Chemical) or for 24 h with trehalose (100 mM, Sigma), rapamycin (300 nM, Sigma), MK2206 (1 μM, Selleckchem), U0126 (10 μM, Tocris) and dialyzed serum (GE Healthcare Life Sciences) for 30 min.

### Cortical and hippocampal neuron cultures

Cortical and hippocampal neurons were prepared from E17.5 and postnatal day 0–1 mice and plated on poly-D-lysine coated six-well plates (BD Biosciences) in Neurobasal medium supplemented with GlutaMAX-I (Invitrogen), B-27 and 1% FBS. At days *in vitro* 4, neurons were treated with 100 mM trehalose. At days *in vitro* 8, neurons were collected and RNA extraction was performed.

### Cortical astrocytes culture

Astrocytes were isolated from P0-1 mice and plated on poly-D-lysine coated six-well plates (BD Biosciences) in the presence of DMEM high glucose, supplemented with 10% FBS and 100 U ml^−1^ penicillin and 100 mg ml^−1^ streptomycin. After 7 days, the glial cell layer was removed and astrocytes were plated for treatment. An amount of 100 mM of trehalose was dissolved in the media the day after and kept for 4 days. Finally, astrocytes were collected and protein extracts were analysed by western blot assay.

### Immunofluorescence assay

For immunofluorescence assay, cells were grown on coverslips in 24-well plates. After the treatment, cells were washed with PBS and fixed with methanol for 10 min. Cells were then blocked with blocking reagent (0.1% saponin, 10% bovine serum in PBS) for 1 h and incubated with appropriate primary antibody(s) (1:100) for 3 h at room temperature. After three washes with PBS, the cells were incubated with appropriate secondary antibodies (1:500) for 1 h at room temperature. Coverslips were then mounted with vectashield containing 4,6-diamidino-2-phenylindole (H-1200) for imaging via confocal microscopy.

### Western blot

Brain tissue and cultured cells were collected and lysed in RIPA buffer (50 mM Tris-HCl, ph 7.4, 1% NP40, 0.5% Na-deoxycholate, 0.1% SDS, 150 mM NaCl, 2 mM EDTA and 50 mM NaF) including a cocktail of protease (Roche) and phosphatase (SIGMA) inhibitors. Protein concentrations were measured with the bicinchoninic acid protein assay kit (Pierce, Rockford, IL), using bovine serum albumin as standard. Lysates were separated via SDS–polyacrylamide gel electrophoresis (PAGE) and then transferred to nitrocellulose membranes. Blots were incubated in blocking buffer (5%, w/v, dried skimmed milk in Tris-buffered saline, pH 7.4 and 0.2% Tween 20, TBST) followed by overnight incubation with appropriate antibodies diluted in blocking buffer (5% dry milk). Western blot images were acquired by LAS 4000 (GE Healthcare) and quantified using ImageJ. Images have been cropped for presentation. Full size images are presented in Supplementary Fig. 18.

### Antibodies

Antibodies to Akt (#9272, 1:1,000), phospho-Akt(S473) (#4060, 1:500), phospho-Akt(T308) (#13038, 1:500), p70 S6K (#9202, 1:1,000), phospho-P70 S6K(T389) (#9205, 1:500), 4E-BP1 (#39452, 1:1,000), phospho-4E-BP1(T37/46) (#9459, 1:500), S6 ribosomal protein (#2217, 1:1,000), phospho-S6 ribosomal protein(S240/244) (#2214, 1:1,000), LAMP1 (#3243, 1:1,000), Histone 3 (#4469, 1:1,000), Phospho-(Ser) 14-3-3 Binding Motif (#9601S, 1:500), Rictor (#2114, 1:500), Raptor (#2280, 1:500), ERK1/2 (#9102, 1:1,000), phosphor-ERK1/2 (#9101, 1:1,000), GSK-3β (D5C5Z) XP (#12456S, 1:1,000), phospho-GSK-3β (Ser9) (#9336S, 1:500), GFP (D5.1) (#29556, 1:1,000) and human TFEB (#4240, 1:500) were purchased from Cell Signaling. Antibody to GAPDH (#32233, 1:1,000) was purchased from Santa Cruz. Antibody to glial fibrillary acidic protein (GFAP) was purchased from DAKO (#Z0334, 1:1,000). Antibody to CD68 was purchased from AbD Serotec (#MCA1957, 1:1,000). Antibody to mouse TFEB was purchased from Proteintech (#13372-1-AP, 1:500). Mouse anti-FLAG M2 (#F1804, 1:1,000) and rabbit anti-FLAG (#F7425, 1:1,000) antibodies were purchased from Sigma. Pan 14-3-3 antibody (K-19) (#SC629, 1:300) was purchased from Santa Cruz. Antibody to TSC2 was purchased from Abcam (#32554, 1:1,000).

### Cytosolic and nuclear protein fractionation

Cell pellets were resuspended in lysis buffer (10 mM Hepes pH 7.9, 10 mM KCl, 0.1 mM EDTA and 0.4% Nonidet P40) with inhibitors by pipetting and kept in ice for 30 min. After 1 min of spin at full speed, the supernatant was collected as cytosolic fraction. The pellet was washed twice with lysis buffer and resuspended with nuclear buffer (20 mM Hepes pH 7.9, 0.4 M NaCl and 1 mM EDTA) containing phosphatases and proteases inhibitors. After 15 min of vigorous shaking on an Eppendorf shaker, the pellet was spun down at full speed for 10 min. The supernatant was used as the nuclear fraction.

### *In vitro* kinase assay

An *in vitro* kinase assay was performed using purified, active AKT1 enzyme (SignalChem, Richmond, Canada). Whole cell lysates for IP were prepared in IP lysis buffer (20 mM Tris, pH 7.5, 150 mM NaCl, 1 mM EDTA and 1% Triton X-100) containing protease inhibitors and 1 mM Na_3_V0_4_. Cell lysates (1,000 μg) were incubated overnight at 4 °C with 10 μg of either mouse anti-FLAG antibody or mouse IgG (Sigma-Aldrich, St. Louis, MO) crosslinked to protein A/G beads (Pierce Crosslink IP Kit, Life Technologies, Grand Island, NY), made up to 300 μl total volume with IP lysis buffer. The immune complexes were collected by centrifugation, washed five times in IP lysis buffer and eluted with 10 μl of 3X FLAG peptide. The eluant was diluted to 30 μl with 1 × kinase buffer (25 mM Tris, pH 7.5, 5 mM β-glycerolphosphate, 10 μM ATP, 2 mM dithiothreitol, 0.1 mM Na_3_VO_4_ and 10 mM MgCl_2_). Kinase reactions were initiated by adding 200 ng of AKT1 and 0.5 μCi [γ-^32^P]ATP (3,000 Ci mmol^−1^, PerkinElmer Life Sciences) in 20 μl of kinase buffer. The reactions were stopped after a 15-min incubation at 30 °C by adding SDS–PAGE loading buffer and heating to 95 °C for 10 min. The samples were resolved on a 4–12% SDS–PAGE gel and analysed by autoradiography. TFEB-S467A-3xFlag was generated by using the QuikChange XLII site-directed mutagenesis kit (Agilent) and the following oligos: 5′-AGCAGCCGCCGGAGCGCCTTCAGCATGGAGGAG-3′, 5-CTCCTCCATGCTGAAGGCGCTCCGGCGGCTGCT-3′, according to the manufacturer’s directions.

### Quantitative real-time PCR

Total RNA was extracted from the control and JNCL fibroblasts and from WT and *Cln3*^*Δex7-8*^ cortical neuron cultures using the RNEasy kit (Qiagen) according to the manufacturer’s instructions. Half of the mouse brain was processed for the RNA extraction and one microgram was used for complementary DNA synthesis by QuantiTect Reverse Transcription kit (Qiagen). The primers for PCR with reverse transcription reactions are listed in Supplementary Table 1. Quantitative real-time PCR was performed by using iQ SYBR Green Supermix on the CFX96 Touch Real-Time Detection System (Bio-Rad Laboratories). Samples were heated for 3 min at 95 °C and amplified in 39 cycles for 11 s at 95 °C, 45 s at 60 °C with last cycle of 10 s at 95 °C, 5 s at 65 °C and 5 s at 95 °C. Analyses were conducted using CFX manager software (Bio-Rad) and the threshold cycle (C_T_) was extracted from the PCR amplification plot. Relative gene expression was determined using the ΔΔC_T_ method, normalizing to *GAPDH* (for human genes) and cyclophilin (for mouse genes). The change in messenger RNA level of the genes was expressed in fold change as previously described^8^. Error bars represent s.e.m. *P<0.05, **P<0.01, ***P<0.001.

### RNA interference

For siRNA knockdown, cells were transfected using Lipofectamine RNAiMAX transfection reagent (Invitrogen) with Stealth RNAi Negative Control Duplex (Thermo-Scientific 12935-300) or with Stealth siRNAs duplex targeted against AKT1 (Thermo Scientific, HSS176614, HSS100346 and HSS100345). siRNA against Rictor was purchased from Cell Signaling (8622). Cells were analysed 72 h after transfection.

### Microarray experiments

Total RNA from control and JNCL fibroblasts with and without trehalose treatment (100 mM, 4 days) was used to prepare complementary DNA for hybridization to the Illumina Human HT-12 V4.0 array platform. Experiments were performed in triplicate. Expression analysis was performed at the Microarray Core and Cell and Regulatory Biology, University of Texas, Houston, TX, USA. A *P*<0.01 was used as a threshold for significance for assessing differential gene expression. GSEA was performed as previously described^10^,^11^. The cumulative distribution function was constructed by performing 1,000 random gene set membership assignments. A nominal *P*<0.01 and an a false discovery rate (FDR)<10% were used as thresholds for significance of the ES. Gene ontology analysis was performed with the web tool DAVID (https://david.ncifcrf.gov/) using default parameters. Pathway co-expression analyses were performed as previously described^8^,^9^, and Cytoscape was used to represent graphically the expression correlation data.

### Animal husbandry

*Cln3*^*Δex7-8*^ mice (stock no. 004685; *Cln3*^*tm1.1Mem*^/J; CD-1 background)^32^ were obtained from the Jackson Laboratory. Control (CD-1) and *Cln3*^*Δex7-8*^ mice were housed 3–4 per cage in a room with a 12-h light/12-h dark cycle. Food and water were provided *ad libitum*. All mice used in this study were analysed at 8 and 12 months of age and were littermates produced by crossing heterozygous *Cln3*^*Δex7-8*^ mice. Only males were used for this analysis. Investigators were blinded when analysing the data, and no randomization was necessary. No data were excluded from this study.

### Intraperitoneal injection

Mice were injected intraperitoneally with MK2206 (120 mg kg^−1^) for four times every other day. MK2206 was formulated in 30% captisol in water. Four *Cln3*^*Δex7-8*^ mice were injected with MK2206 and four were injected with 30% captisol as vehicle control.

### Trehalose treatment

Trehalose (Swanson) was dissolved in drinking water to a final concentration of 2% and changed twice a week. Trehalose-containing water was given to *Cln3*^*Δex7-8*^ and WT mice by spontaneous oral administration starting at 21 days of age and continuing until the day the mice died naturally (life span assessment) or were sacrificed for other studies.

### Immunohistochemistry

Eight- and 12-month-old homozygous *Cln3*^*Δex7-8*^ mice and age-matched controls were anaesthetized with isoflurane and transcardially perfused with PBS followed by 4% buffered paraformaldehyde in 0.1 M sodium phosphate buffer, pH 7.4. Brains were subsequently removed and postfixed overnight. Before sectioning, the brains were cryoprotected in a solution containing 30% sucrose in Tris-buffered saline (TBS: 50 mM Tris, pH 7.6). Consecutive 40 μm floating coronal sections were collected in 96-well plates. Series of sections were then stained with primary antisera against CD68 or GFAP, followed by either rabbit anti-rat (VectorLab) and swine anti-rabbit (DAKO) secondary antibodies, and immunoreactivity detected with Vectastain ABC (avidin-biotin) kit (Vector) and diaminobenzidine as a chromogen.

### Quantitative analysis of glial phenotype

Thirty non-overlapping images were captured, on three consecutive sections, through each region of interest. All RGB images were captured via a live video camera (JVC, 3CCD, KY-F55B), mounted onto a Zeiss Axioplan microscope using a × 40 objective and saved as JPEGs. All parameters including lamp intensity, video camera set-up and calibration were maintained constant throughout image capturing. Images were subsequently analysed using ImageJ analysis software (NIH), using an appropriate threshold that selected the foreground immunoreactivity above background. This threshold was then applied as a constant to all subsequent images analysed per batch of animals and reagent used to determine the specific area of immunoreactivity for each antigen in each region. This analysis was performed blind to genotype. Data were plotted graphically as the mean percentage area of immunoreactivity per field±s.e.m. for each region.

### Storage burden

To analyse the relative level of the autofluorescent storage material present in each brain region, mouse brain sections spanning the S1BF and VPM/VPL were mounted onto gelatin-chrome-coated slides and cover-slipped with Vectashield (Vector Laboratories, Peterborough, UK). Non-overlapping images from each section were captured at × 63 magnification using a Leica SP5 confocal microscope and a 488 nm excitation laser (Leica Microsystem). Thresholding image analysis was performed to determine the storage burden present in each region. During image capture, all laser parameters and calibrations were kept constant. Semiquantitative thresholding image analysis was carried out using ImageJ (NIH).

### TEM

Mice were anaesthetized and perfused intracardially with saline solution followed by 2% formaldehyde+2.5% glutaraldehyde in 0.1 M sodium cacodylate buffer (pH 7.4). Brains were removed and small pieces of cerebellum and cortex were collected, and further postfixed in 2% formaldehyde+2.5% glutaraldehyde, 0.1 M sodium cacodylate buffer (pH 7.4) for 24 h. One-hundred micrometre coronal sections were cut with a vibratome and fixed in 1% OsO_4_ in 0.1 M cacodylate for 1 h, stained with uranyl acetate dehydrated and embedded in Eponate 812. Ultrathin sections at 60 nm were obtained on an RMC MT6000 ultramicrotome and examined with a Hitachi H7500 transmission electron microscope. Images were captured using a Gatan US1000 high-resolution digital camera and Digital Micrograph software (v1.82.366).

### Tissue preparation for MRI

Mice were transcardially perfused before imaging. The head was removed and then the skin, muscle, ears, nose tip and lower jaw were removed to expose the skull. The head was fixed overnight in 4% paraformaldehyde at 4 °C. The head was then transferred to 40 mLs of 0.01% sodium azide in PBS and rocked for 7 days at 4 °C. The head was transferred to a solution of 5 mM gadopentetate dimeglumine (Bayer HealthCare Pharmaceuticals Inc., Wayne, NJ) and 0.01% sodium azide in PBS and rocked for 25–35 days at 4 °C. Incubation with gadopentetate dimeglumine improved the signal-to-noise ratio. Before imaging, the head was equilibrated to room temperature for 6–8 h.

### Magnetic resonance protocol

A total of 48 scans per mouse were acquired on a 9.4 T Bruker Avance Biospec Spectrometer, 21-cm bore horizontal scanner with 35 mm volume resonator (Bruker BioSpin, Billerica, MA) with Paravision 5.0 software (Bruker Biospin, Billerica, MA). The three-dimensional diffusion tensor imaging (DTI) scan parameters are as follows: spin echo, *b*-value=0 and 1,000 s mm^−2^, 20 diffusion directions with one non-diffusion weighted image, repetition time (TR)=500 ms, echo time (TE)=14.8 ms, field of view (FOV)=1.7 × 1.2 × 2.4 cm or 2.0 × 1.4 × 3.2 cm, matrix=128 × 96 × 96, number of excitations (NEX)=1, *δ*=3 ms, Δ=7 ms. The acquisition time was ∼15 h.

### MRI image processing

The MRI images were first processed on DTI studio to extrapolate the fractional anisotropy. Subsequently, the Amira software (Visage Imaging, Inc., San Diego, CA) was used to define the ROI of the CC and to calculate the volume for each mouse. Volumetric measurements of the CC were performed in a blinded manner.

### ABR measurements

ABRs were measured as previously described^69^. Briefly, 10-month-old mice (*n*=4–6 per genotype/treatment group) were anaesthetized using an intraperitoneal injection of ketamine (100 mg kg^−1^) and xylazine (10 mg kg^−1^) and then immobilized in a head holder. Normal body temperature was maintained throughout the procedure by placing the mice on a heating pad. Pure tone stimuli from 4 to 48 kHz were generated using Tucker-Davis Technologies System 3 digital signal processing hardware and software (Tucker-Davis Technologies, Alachua, FL, USA), and the intensity of the tone stimuli was calibrated using a type 4,938 1/4″ pressure-field calibration microphone (Bruel and Kjar, Nærum, Denmark). Response signals were recorded with subcutaneous needle electrodes inserted at the vertex of the scalp, the postauricular region and the back leg (ground)^69^. Auditory thresholds were determined by decreasing the sound intensity of each stimulus from 90 to 10 dB in 5 dB steps, until the lowest sound intensity with reproducible and recognizable waves in the response was reached. Mean hearing thresholds±s.d. (dB sound pressure level (SPL)) were plotted as a function of stimulus frequency (kHz). Statistical analysis consisted of one-way analyses of variance to reveal overall trends accompanied by two-tailed Student’s *t*-tests at individual frequencies to evaluate frequency-specific effects. *T*-test *P* values were adjusted for multiple comparisons using the Holm method. R (version 2.13) was used for all statistical analyses.

### Akt phosphosite prediction

To identify candidate phosphosites that may be targeted by Akt, experimentally determined, non-redundant Akt phosphosite sequences were downloaded from PhosphositePlus website (http://www.phosphosite.org/) and used to build a PWM to scan TFEB amino-acid sequence using the MEME Suite 4.11.0 (http://meme-suite.org/). TFEB sequences were aligned by using MultAlin (http://multalin.toulouse.inra.fr/) with default parameters.

### Statistics

The results are presented as the means±s.e.m. Statistical significance of mean differences for each parameter was determined by analysis of variance for genotype and treatment followed by Tukey’s *post hoc* test unless otherwise indicated. A *P*<0.05 was considered significant.

### Study approval

All mouse experimental procedures were reviewed and approved by the Institutional Animal Care and Use Committee at Baylor College of Medicine.

### Data availability

The authors declare that all data supporting the findings of this study are available within the article and its Supplementary Information files. The Gene Expression Omnibus accession number for gene expression microarray is GSE76643.

## Additional information

**How to cite this article:** Palmieri, M. *et al*. mTORC1-independent TFEB activation via Akt inhibition promotes cellular clearance in neurodegenerative storage diseases. *Nat. Commun.* **8,** 14338 doi: 10.1038/ncomms14338 (2017).

**Publisher’s note:** Springer Nature remains neutral with regard to jurisdictional claims in published maps and institutional affiliations.

## Supplementary Material

Supplementary Information Supplementary Figures and Supplementary Table.

Supplementary Movie 1 3D reconstruction of the corpus callosum from an untreated Cln3Δex7-8 mouse. Reconstruction is based on 48 scans obtained from a 12-month-old JNCL mouse by MRI analysis.

Supplementary Movie 2 3D reconstruction of the corpus callosum from an untreated WT mouse. Reconstruction is based on 48 scans obtained from a 12-month-old wild-type mouse by MRI analysis.

Supplementary Movie 3 3D reconstruction of the corpus callosum from a treated Cln3Δex7-8 mouse. Reconstruction is based on 48 scans obtained by MRI analysis of a 12-month-old JNCL mouse that was treated with trehalose from weaning.

Supplementary Movie 4 3D reconstruction of the corpus callosum from a treated WT mouse. Reconstruction is based on 48 scans obtained by MRI analysis of a 12-month-old wildtype mouse that was treated with trehalose from weaning.
