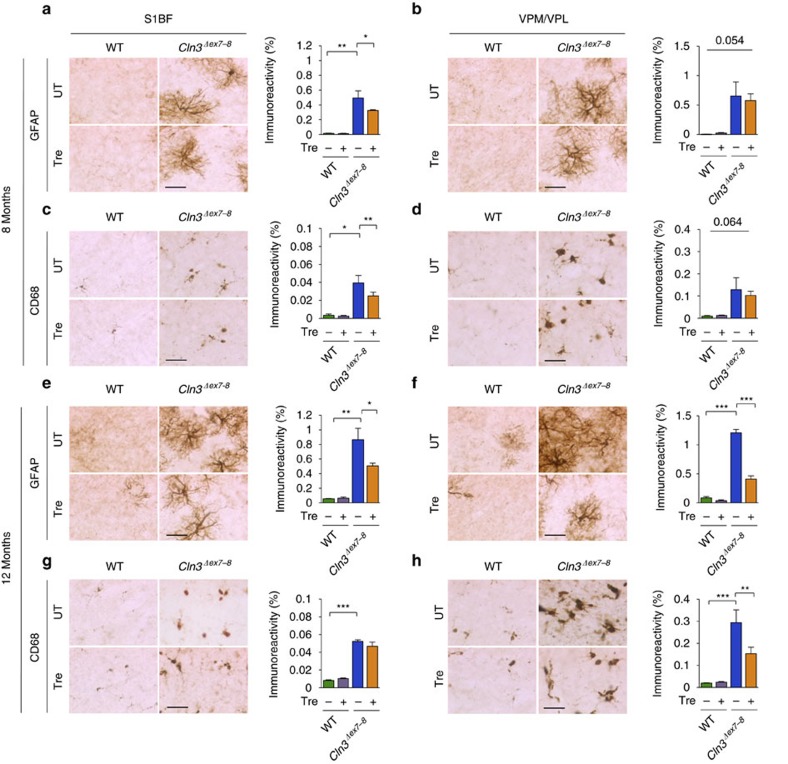# Corrigendum: mTORC1-independent TFEB activation via Akt inhibition promotes cellular clearance in neurodegenerative storage diseases

**DOI:** 10.1038/ncomms15793

**Published:** 2017-06-13

**Authors:** Michela Palmieri, Rituraj Pal, Hemanth R. Nelvagal, Parisa Lotfi, Gary R. Stinnett, Michelle L. Seymour, Arindam Chaudhury, Lakshya Bajaj, Vitaliy V. Bondar, Laura Bremner, Usama Saleem, Dennis Y. Tse, Deepthi Sanagasetti, Samuel M. Wu, Joel R. Neilson, Fred A. Pereira, Robia G. Pautler, George G. Rodney, Jonathan D. Cooper, Marco Sardiello

Nature Communications 8: Article number: 14338; DOI: 10.1038/ncomms14338 (2017); Published: 02 06 2017; Updated: 06 13 2017

This Article contains errors in Figs 2 and 3, for which we apologize. In Fig. 2c, the four images were inadvertently duplicated from the images in Fig. 2b. In Fig. 3g, the image at the upper right corner, corresponding to the condition UT_ C*ln3*^*Δex7-8*^ was inadvertently duplicated from the image in the lower right corner of Fig. 3d. The correct versions of these figures appear below as Figs 1 and 2 respectively. The raw data associated with these experiments is provided as a separate Supplementary Data file.

## Supplementary Material

Supplementary Information

## Figures and Tables

**Figure 1:**
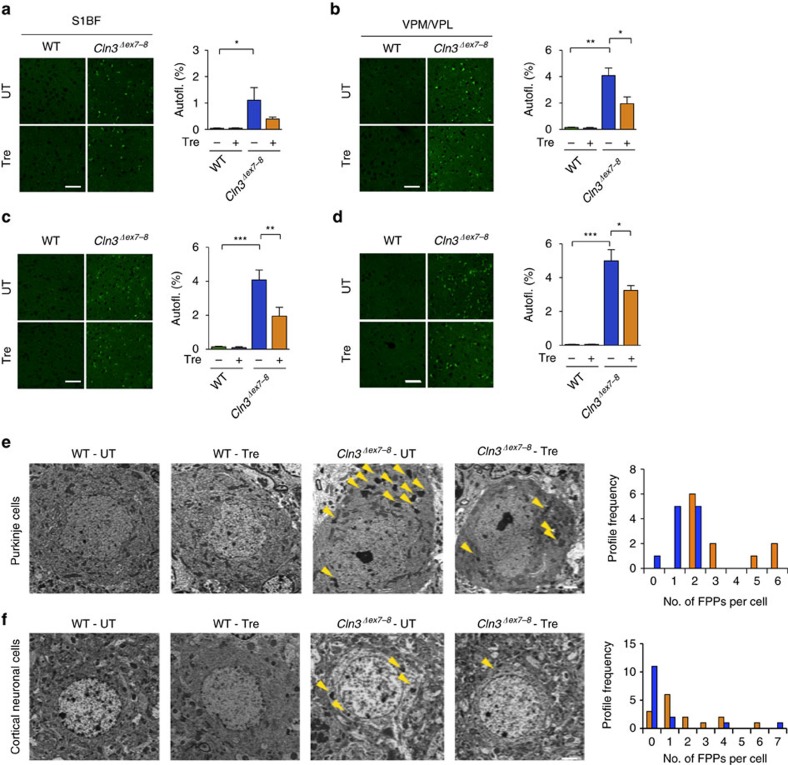


**Figure 2:**