# Supplementary material for: mTORC1-independent TFEB activation via Akt inhibition promotes cellular clearance in neurodegenerative storage diseases
Source: Nat Commun. 2017 Feb 6;8:14338. doi: 10.1038/ncomms14338 (PMC5303831; doi:10.1038/ncomms14338)
Supplement: Supplementary Information — Supplementary Figures and Supplementary Table. [file ncomms14338-s1.pdf]

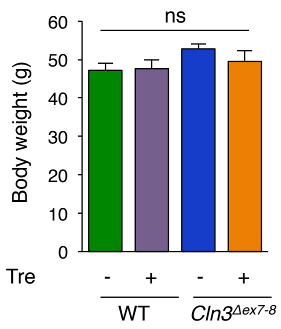

Supplementary Figure 1. Assessment of body weight in treated and untreated mice. Histogram of the body weight of 12-month-old WT and *Cln3*$^{\Delta ex7\text{-}8}$ mice reveals no differences between genotypes irrespective of trehalose (Tre) treatment. ns, not significant. All groups of mice, n = 8 to 11. Data represent means ± SEM.

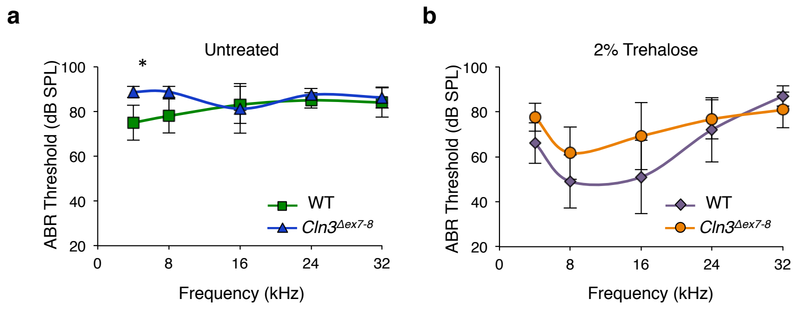

Supplementary Figure 2. Assessment of hearing function in treated and untreated mice. (**a**) Auditory brainstem responses (ABR) at 10 months of age show elevated ABR thresholds in *Cln3*$^{\Delta ex7\text{-}8}$ mice compared to WT littermates, indicative of hearing loss. (**b**) Trehalose treatment reduced ABR thresholds in both genotypes, indicative of improved hearing. All groups of mice, *n* = 4 to 6. Data represent means ± SEM. *$P < 0.05$.

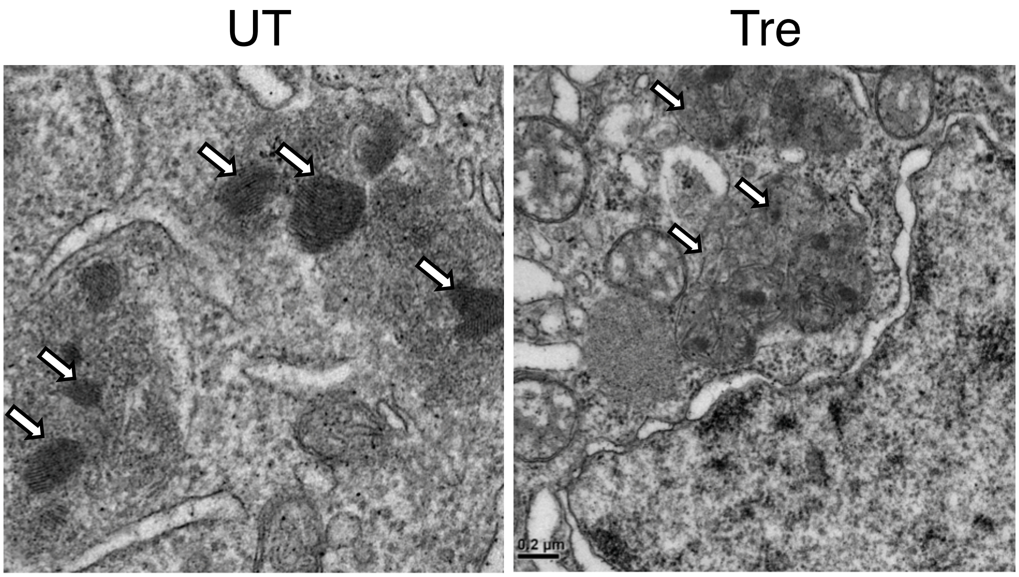

Supplementary Figure 3. Transmission electron microscopy of lysosomal storage burden at 12 months of age in treated and untreated *Cln3*$^{\Delta ex7\text{-}8}$ mice. Electron micrographs show the presence of finger print profiles (FPPs) in the lysosomes of untreated JNCL mice which are dramatically reduced in the treated mice. The micrographs are representative examples of Purkinje cells from the cohorts of untreated (UT) and treated (Tre) mice. Arrows indicate FPPs. Scale bar is 0.2 µm.

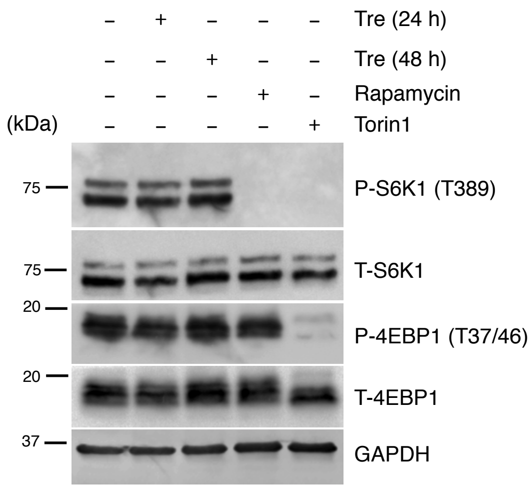

Supplementary Figure 4. Trehalose does not alter mTORC1 activity. HeLa cells were treated with trehalose for 24 h or 48 h, or with rapamycin (600 nM, 16 h) or Torin1 (300 nM, 2 h) as controls for mTORC1 inhibition. Immunoblot analyses of mTORC1 substrates show no changes in their phosphorylation state upon trehalose treatment. GAPDH was used as a loading control.

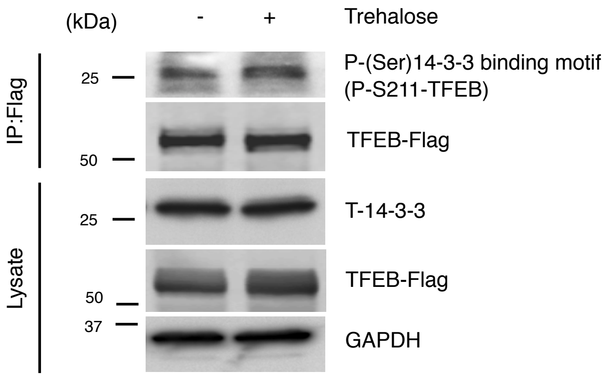

Supplementary Figure 5. Trehalose does not modify phosphorylation of TFEB at S211. TFEB-Flag was immunoprecipitated from HeLa cells transfected with TFEB-Flag and treated with trehalose for 24 h or left untreated. Immunoblot analyses were performed using antibody against Phospho(Ser)-14-3-3 binding motif and control antibodies.

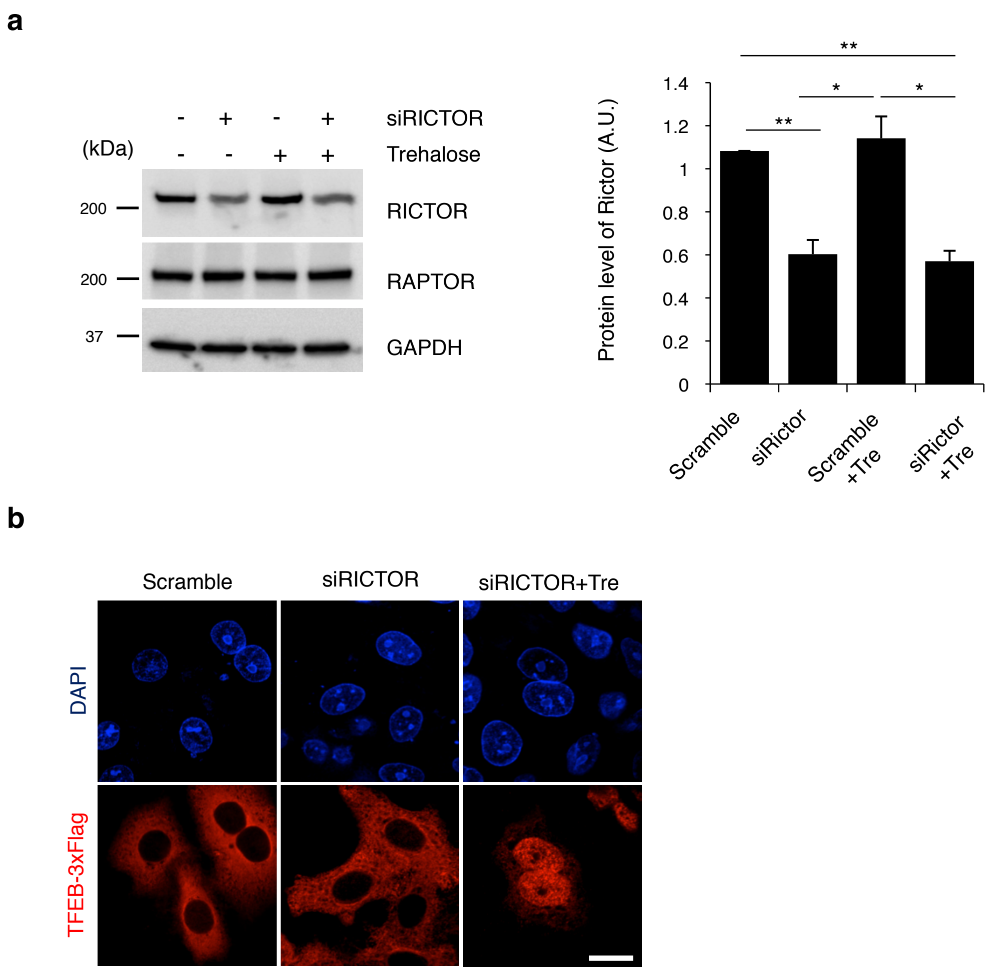

Supplementary Figure 6. TFEB subcellular localization is independent of mTORC2. (**a**) HeLa cells were transfected with siRNA against Rictor for 72 h where indicated. Cells were treated with trehalose for 24 h before of analysis where indicated. The bar diagram represents average values from three replicates. Data represent means ± SEM. $*P < 0.05$, $**P < 0.001$ (**b**) HeLa/TFEB-Flag cells were treated as in (**a**) and labeled for immunofluorescence confocal analysis. Scale bar is 20 μm.

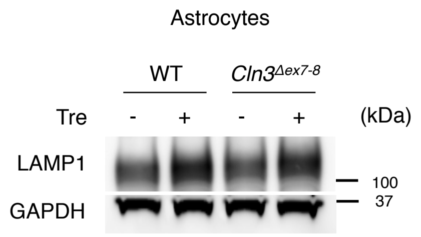

Supplementary Figure 7. Lysosomal enhancement in treated astrocytes from WT and *Cln3$^{\Delta ex7\text{-}8}$* mice. Immunoblot analysis of the lysosomal marker, Lamp1, on cultured astrocytes isolated from wild-type (WT) and JNCL (*Cln3$^{\Delta ex7\text{-}8}$*) mice.

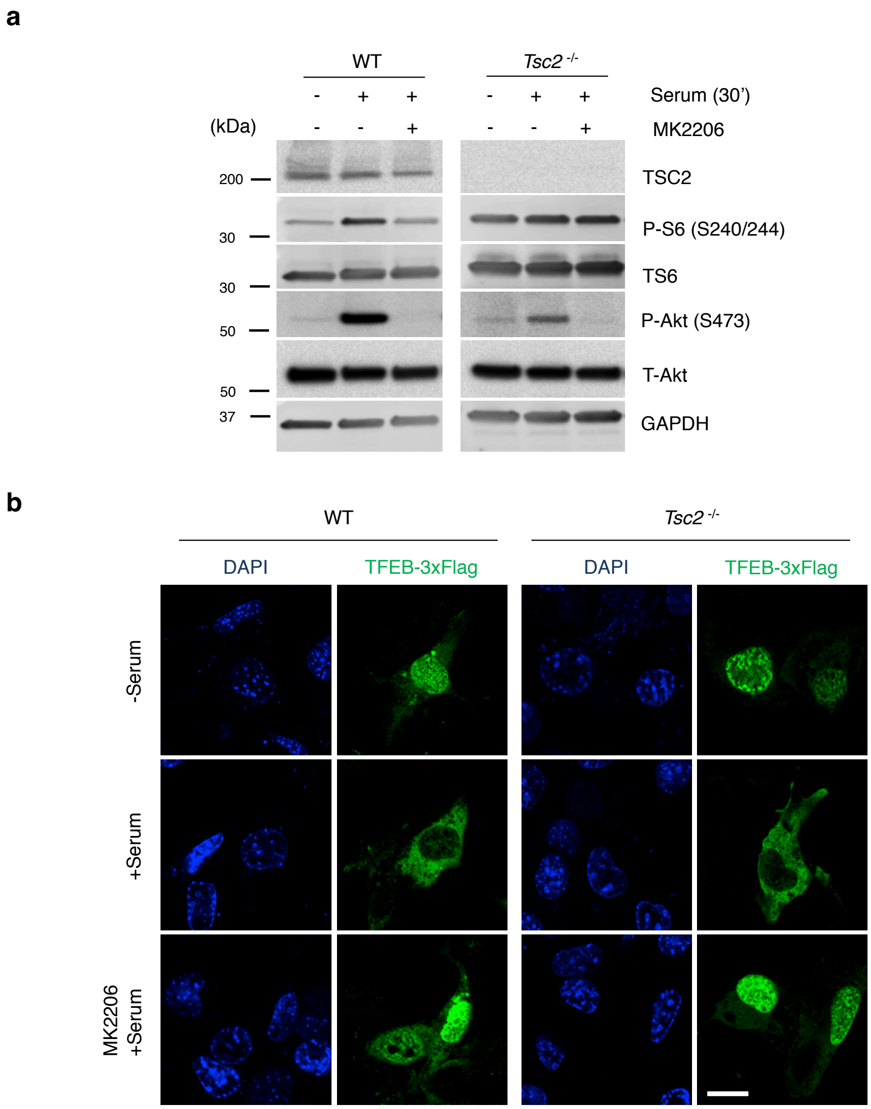

Supplementary Figure 8. Serum stimulation modulates subcellular localization of TFEB by regulating Akt activity. (**a**) WT and *Tsc2*$^{-/-}$ cells were serum starved (16 h), treated with MK2206 in the last two hr of starvation where indicated, and stimulated with dialyzed serum for the last 30 min when indicated. Cell lysates were probed with antibodies as indicated. (**b**) WT and *Tsc2*$^{-/-}$ cells were transiently transfected with TFEB-Flag and treated as in (**a**) and analyzed by immunofluorescence confocal microscopy. Scale bar is 60 μm.

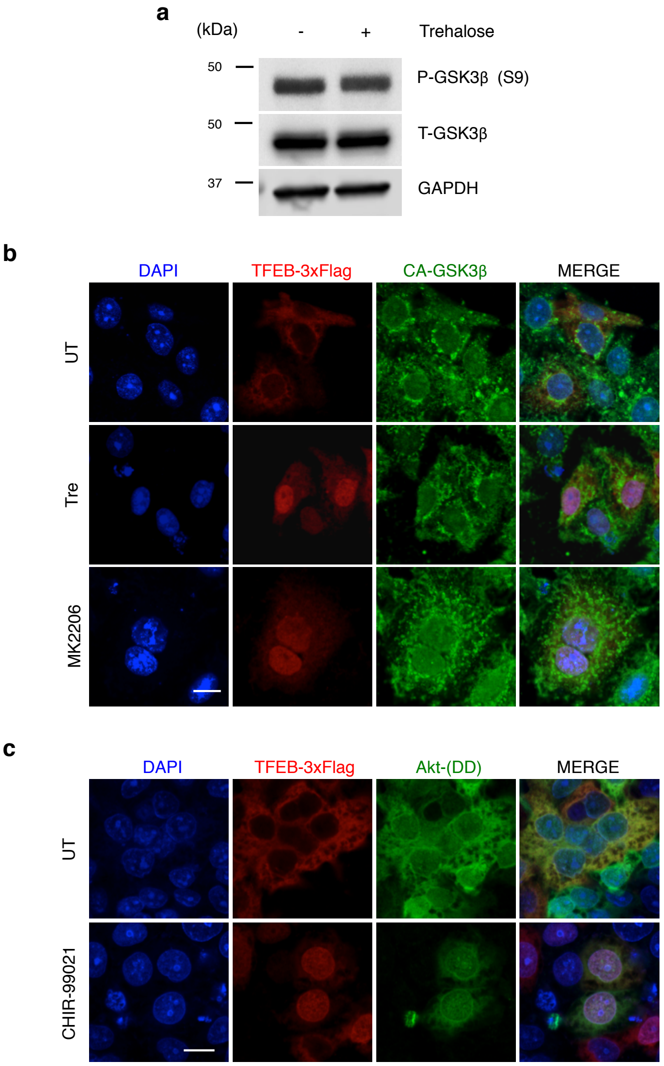

Supplementary Figure 9. Trehalose controls Akt regulation of TFEB in a GSK3β-independent manner. (**a**) HeLa cells were treated with trehalose for 24 h or left untreated. Immunoblot analyses were used to evaluate levels of GSK3β and its phosphorylation status. GAPDH was used as a loading control. (**b**) HeLa cells were cotransfected with TFEB-3xFlag and constitutively active GSK3β (CA-GSK3β), treated with trehalose or MK2206 for 24 h, and examined by immunofluorescence labeling for Flag (red) and GSK3β (green). Scale bar is 20 μm. (**c**) HeLa cells were cotransfected with TFEB-3xFlag and constitutively active Akt (Akt-DD), treated with the GSK3β inhibitor CHIR99021 for 24 h, and examined by immunofluorescence labeling for Flag (red) and Akt (green). Scale bar is 20 μm.

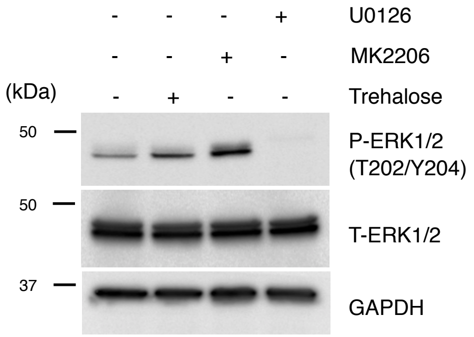

Supplementary Figure 10. Trehalose does not inhibit ERK. HeLa cells were treated with trehalose, MK2206 (Akt inhibitor) or U0126 (ERK inhibitor) for 24 h. Immunoblot analyses were used to evaluate levels of ERK and its phosphorylation status. GAPDH was used as a loading control.

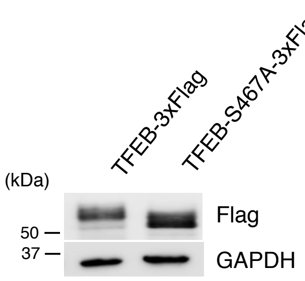

Supplementary Figure 11. Shift of molecular weight of a S467A TFEB version. Western blot analysis of total protein extracts from HeLa cells that were transiently transfected with TFEB-Flag or TFEB-S467A-Flag plasmids shows a shift of TFEB-S467A to a lower molecular weight.

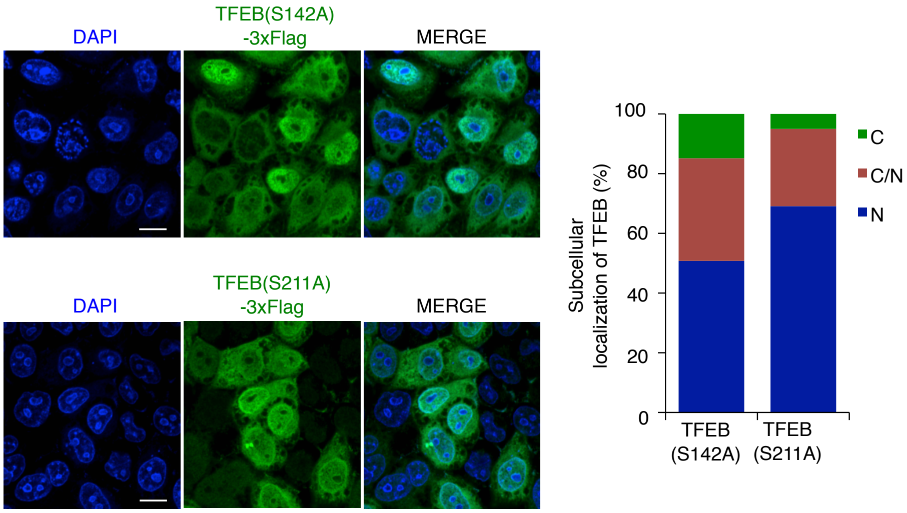

Supplementary Figure 12. Confocal microscopic analysis of TFEB-S142A and TFEB-S211A. HeLa cells were transiently transfected with the indicated constructs and analyzed by immunofluorescence confocal microscopic analysis. Scale bar is 10 μm.

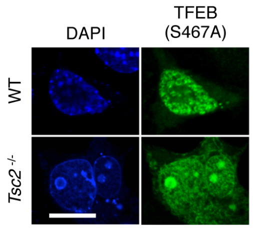

Supplementary Figure 13. TFEB(S467A) nuclear localization in WT and *Tsc2*$^{-/-}$ mouse embryonic fibroblasts. WT and *Tsc2*$^{-/-}$ MEFs were transiently transfected with TFEB(S467A) and analyzed by confocal microscopy. Scale bar is 10 μm.

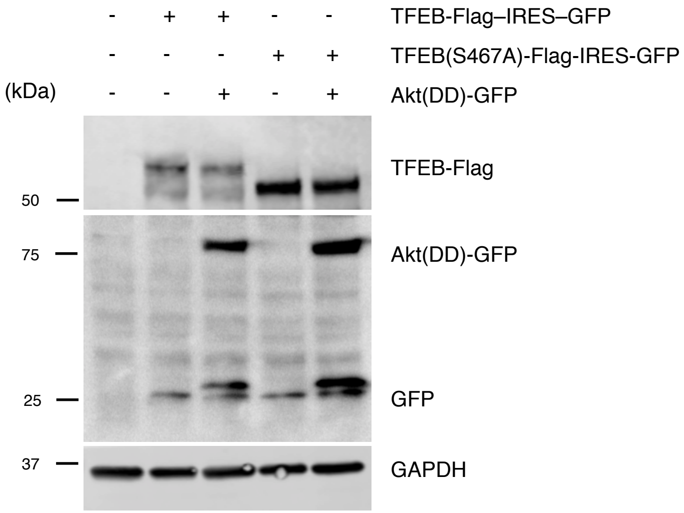

Supplementary Figure 14. Akt regulates TFEB stability. Immunoblot of lysates from cells co-transfected with bicistronic TFEB-Flag–IRES–GFP or TFEB(S467A)-Flag–IRES–GFP with and without Akt(DD)-GFP vectors showing that the mutant TFEB protein is more stable than wild-type TFEB.

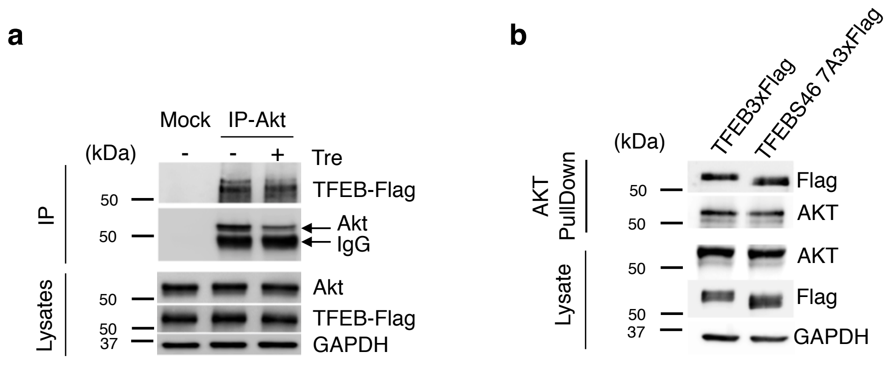

Supplementary Figure 15. Akt interacts with TFEB. (**a**) Co-immunoprecipitation assay showing TFEB interaction with Akt. (**b**) Substitution of TFEB Ser467 with Ala does not affect the binding with Akt.

a

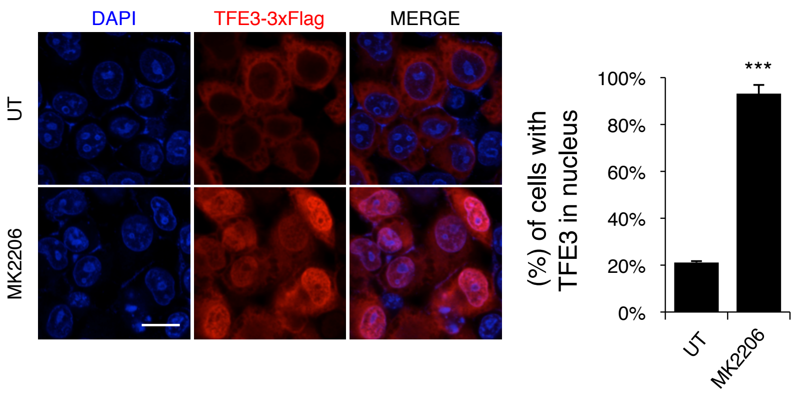

b

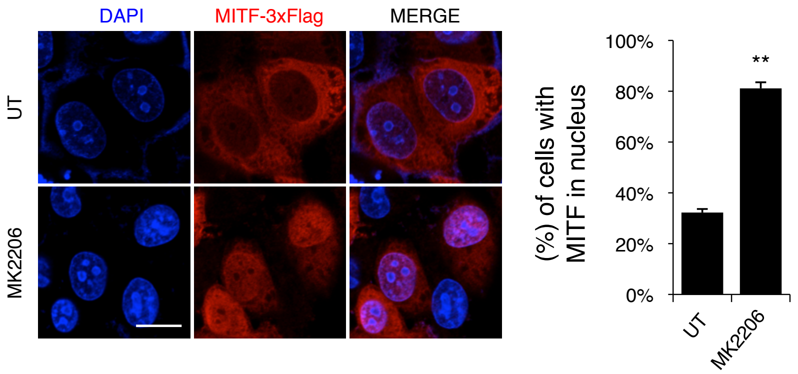

Supplementary Figure 16. Pharmacological inhibition of AKT induces nuclear translocation of TFE3 and MITF. HeLa cells were transiently transfected with TFE3-3xFlag (**a**) and MITF-3xFlag (**b**) and analyzed by confocal microscopy. Scale bar is 10 μm.

a

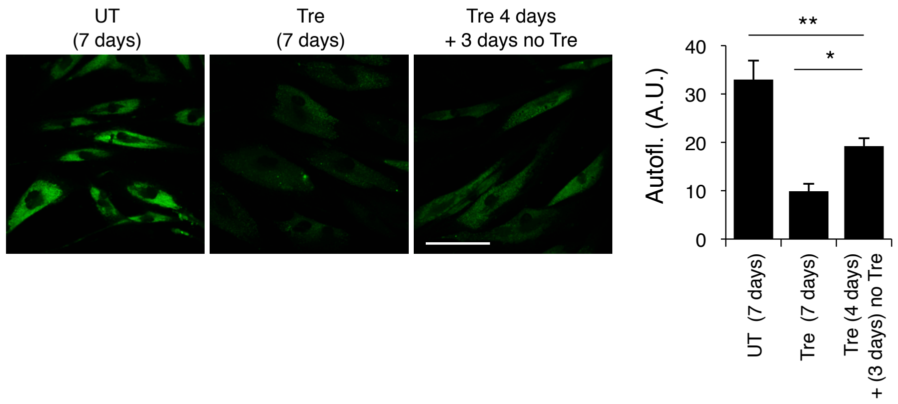

b

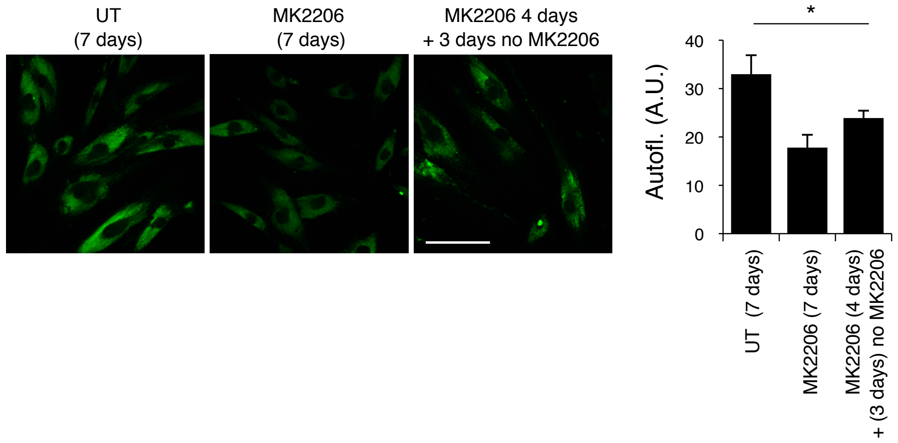

Supplementary Figure 17. Effect of trehalose and Akt on intralysosomal ceroid lipopigment storage. Confocal microscopy analysis of primary fibroblasts with defective CLN3 (c.461-677del). (**a**) Cells were treated with trehalose for 7 days or for 4 days followed by removal of trehalose, and let grow for another 3 days. (**b**). Cells were treated with MK2206 for 7 days for 4 days followed by removal of MK2206, and let grow for another 3 days. Data represent means ± SEM. *$P < 0.05$, **$P < 0.01$. Scale bar is 30 μm.

Supplementary Figure 18

Fig. 4c

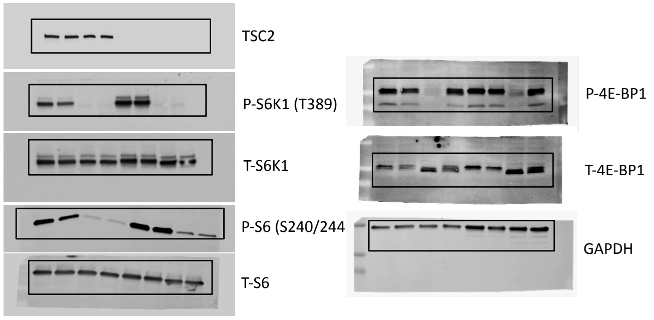

Fig. 7b

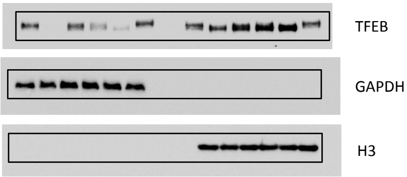

Fig. 7g

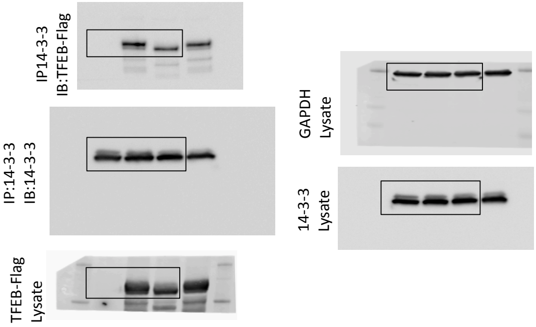

Fig. 7h

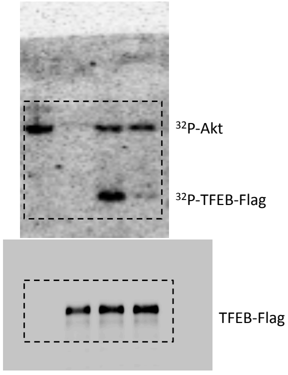

Fig. 7i

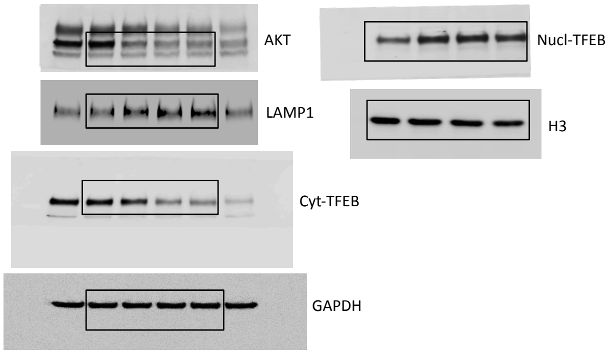

Fig. 7j

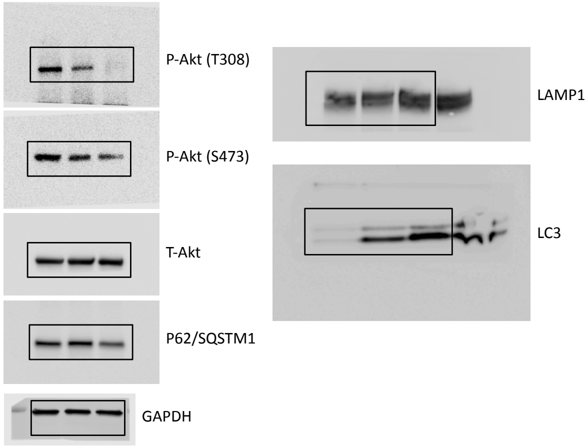

Fig. 7l

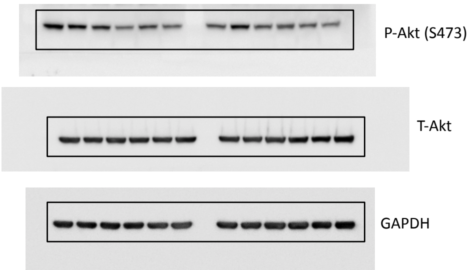

Fig. 8b

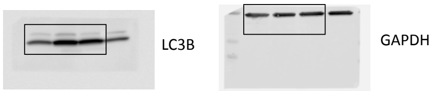

Supplementary Figure 18 - continued

Fig. 8e

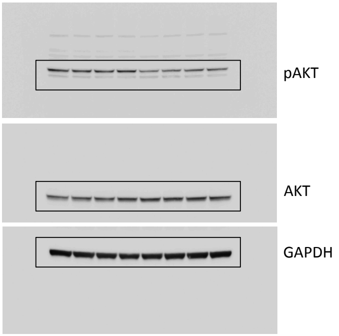

Suppl. Fig. 4

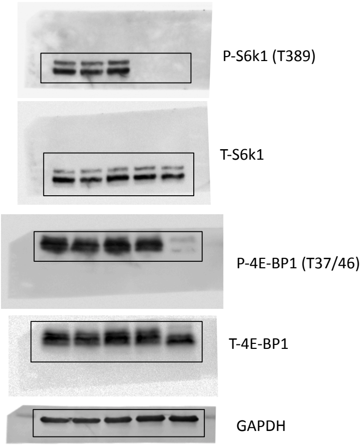

Suppl. Fig. 5

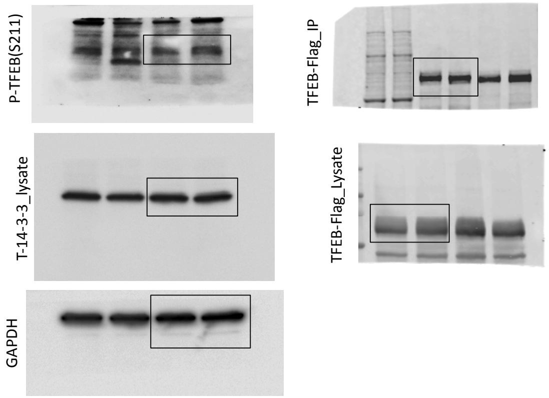

Suppl. Fig. 6a

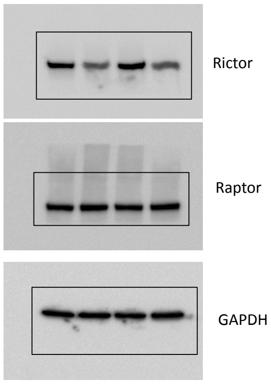

Suppl. Fig. 7

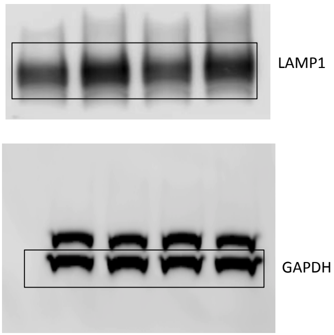

Suppl. Fig. 8a

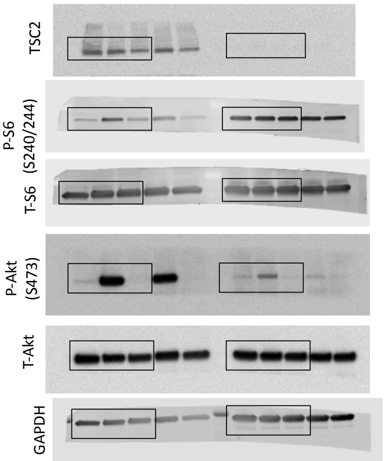

Supplementary Figure 18 - continued

Suppl. Fig. 9a

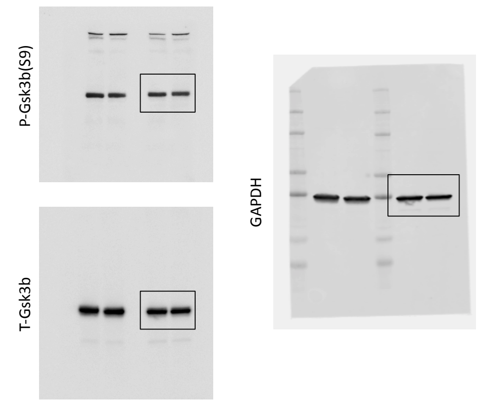

Suppl. Fig. 10

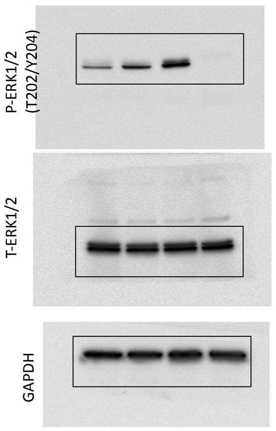

Suppl. Fig. 11

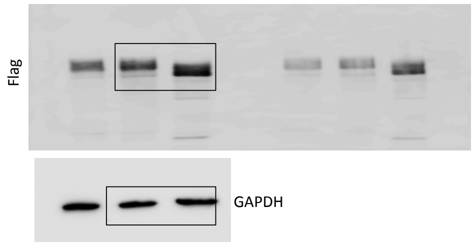

Suppl. Fig. 14

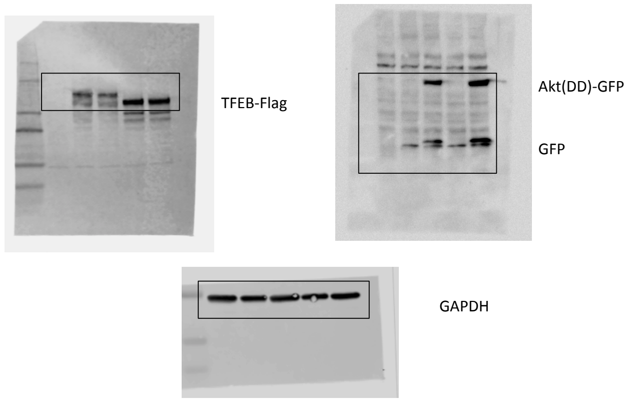

Suppl. Fig. 15

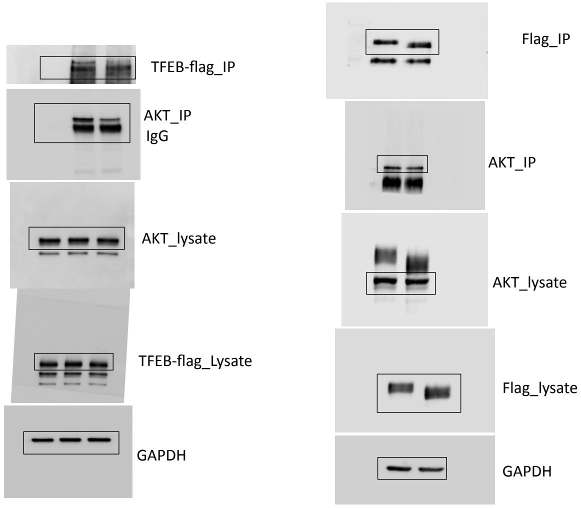

Supplementary Figure 18. Full scans of Western blots shown in Figures 4, 7, 8, and Supplementary Figures 4, 5, 6, 7, 8, 9, 10, 11, 14, 15.

Supplementary Table 1. Sequences of oligos used in real-time qPCR analysis

Gene name	Forward oligos	Reverse oligos
	Human genes	
CTSA	CAGGCTTTGGTCTTCTCTCCA	TCACGCATTCCAGGTCTTTG
CTSD	AACTGCTGGACATCGCTTGCT	CATTCTTCACGTAGGTGCTGGA
HEXA	CAACCAACACATTCTTCTCCA	CGCTATCGTGACCTGCTTTT
MCOLN1	TTGCTCTCTGCCAGCGGTACTA	GCAGTCAGTAACCACCATCGGA
SGSH	TGACCGGCCTTTCTTCCTCTA	GCTCTCTCCGTTGCCAAACTT
SQSTM1	AAGCTGCCTTGTACCCAC	CGCTCCGATGTCATAGTTCTTG
BECLIN	AAGAGGTTGAGAAAGGCGAG	TGGGTTTTGATGGAATAGGAGC
TPP1	GATCCCAGCTCTCCTCAATACG	GCCATTTTTGCACCGTGTG
UVRAG	CATCTGTGTCTTGTTTCGTGG	TTCATTTTGGTTTCGGGCATG
MAP1LC3B	AGCAGCATCCAACCAAAATC	CTGTGTCCGTTCACCAACAG
GAPDH	TGCACCACCAACTGCTTAGC	GGCATGGACTGTGGTCATGAG
APRT	CACTCTGTGGGCCTGGTATT	CTCCAGGGCGTCTTTCTGAA
	Mouse genes	
Ctsa	TTCTGATCCAGCCAGATGGTG	TACAGCACGTTGGCAATCAGG
Gaa	CTCCTACCCAGGTCCTTTCCAA	ATGGCCAGGCTCTTGTTGTCAG
Glb1	AAATGGCTGGCAGTCCTTCTG	ACCTGCACGGTTATGATCGGT
Ctsd	CGTCCTTTGACATCCACTACGG	TGGAACCGATACAGTGTCCTGG
Gns	ACCTGACAGATGTTCTGGCCA	CGCTGGAGTGGAGATCATCAT
Mcoln1	GCGCCTATGACACCATCAA	TATCCTGGCACTGCTCGAT
Sgsh	CCTGCTGCACAATTCTGTTGG	TCCGTCATCCGCAACTATCAG
Tcfeb	GTCATTGACAACATTATGCGCC	GCGTGTTAGGCATCTTGCATCT
Lamp1	CCTACGAGACTGCGAATGGT	CCACAAGAACTGCCATTTTTC
Gaa	CTCCTACCCAGGTCCTTTCCAA	ATGGCCAGGCTCTTGTTGTCAG
Map1lc3b	GCTTGCAGCTCAATGCTAAC	CCTGCGAGGCATAAACCATGTA
Sqstm1	GAAGCTGCCCTATACCCACA	TGGGAGAGGGACTCAATCAG
Ambra	GAGCACCCAATTTACCCAGA	GATCATCCTCTGGGCGTAGTA
Beclin	AGGCTGAGGCGGAGAGATT	TCCACACTCTTGAGTTCGTCAT
Gabarap	CAAAGAGGAGCATCCGTTCGAG	TTGTCCAGGTCTCCTATCCGAG
Tpp1	CCCCTCATGTGGATTTTGTGG	TGGTTCTGGACGTTGTCTTGG
Uvrag	CAAGCTGACAGAAAAGGAGCGAG	GGAAGAGTTTGCCTCAAGTCTGG
Cyclophilin	GGCAAATGCTGGACCAAACACAA	GTAAAATGCCCGCAAGTCAAAAG
S16	AGGAGCGATTTGCTGGTGTGG	GCTACCAGGGCCTTTGAGATG
