# Supplementary material for: Corrigendum: mTORC1-independent TFEB activation via Akt inhibition promotes cellular clearance in neurodegenerative storage diseases
Source: Nat Commun. 2017 Jun 13;8:15793. doi: 10.1038/ncomms15793 (PMC5474731; doi:10.1038/ncomms15793)

Raw images for Figure 2c

WT, UT

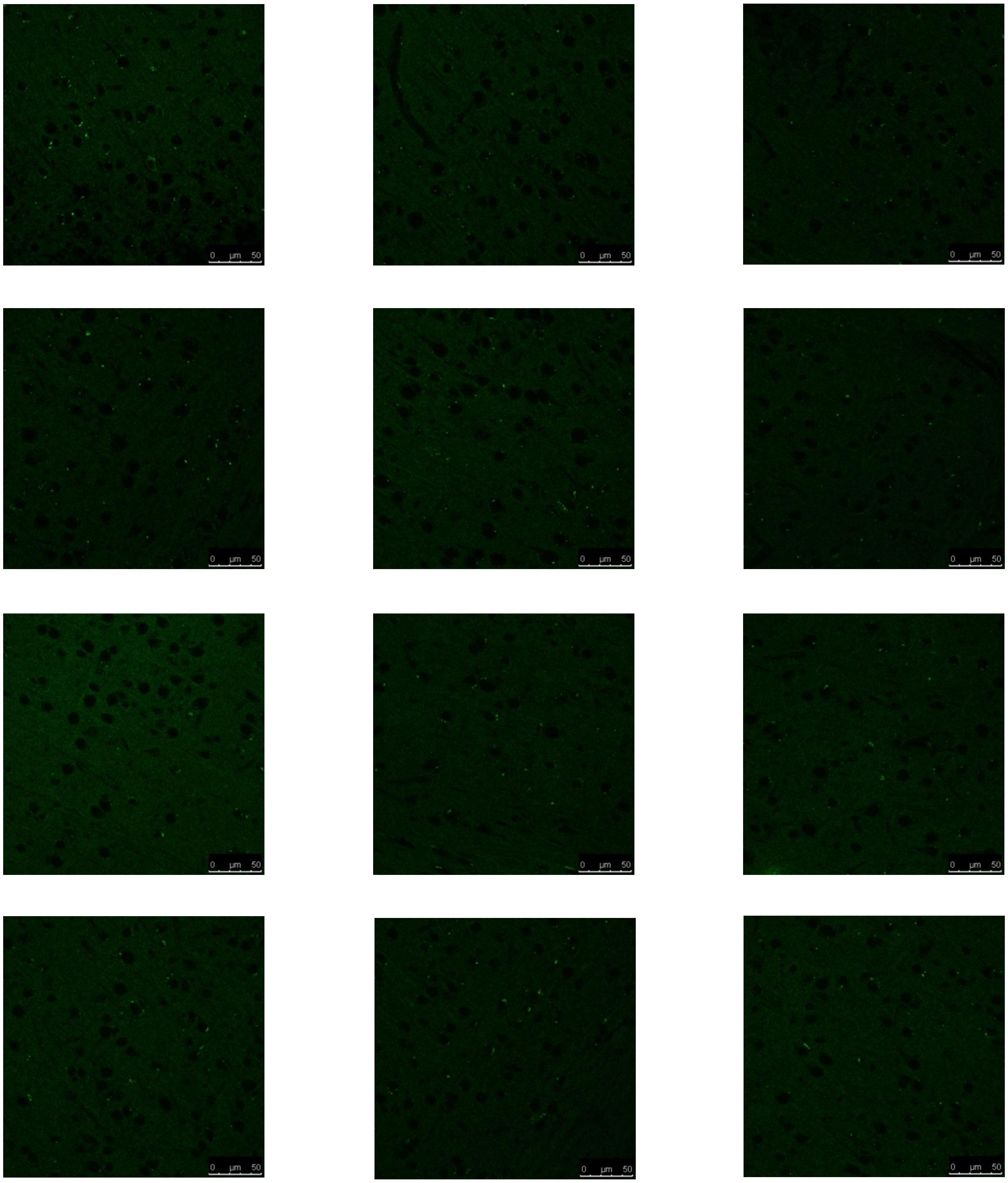

Raw images for Figure 2c (continued)

WT, Tre

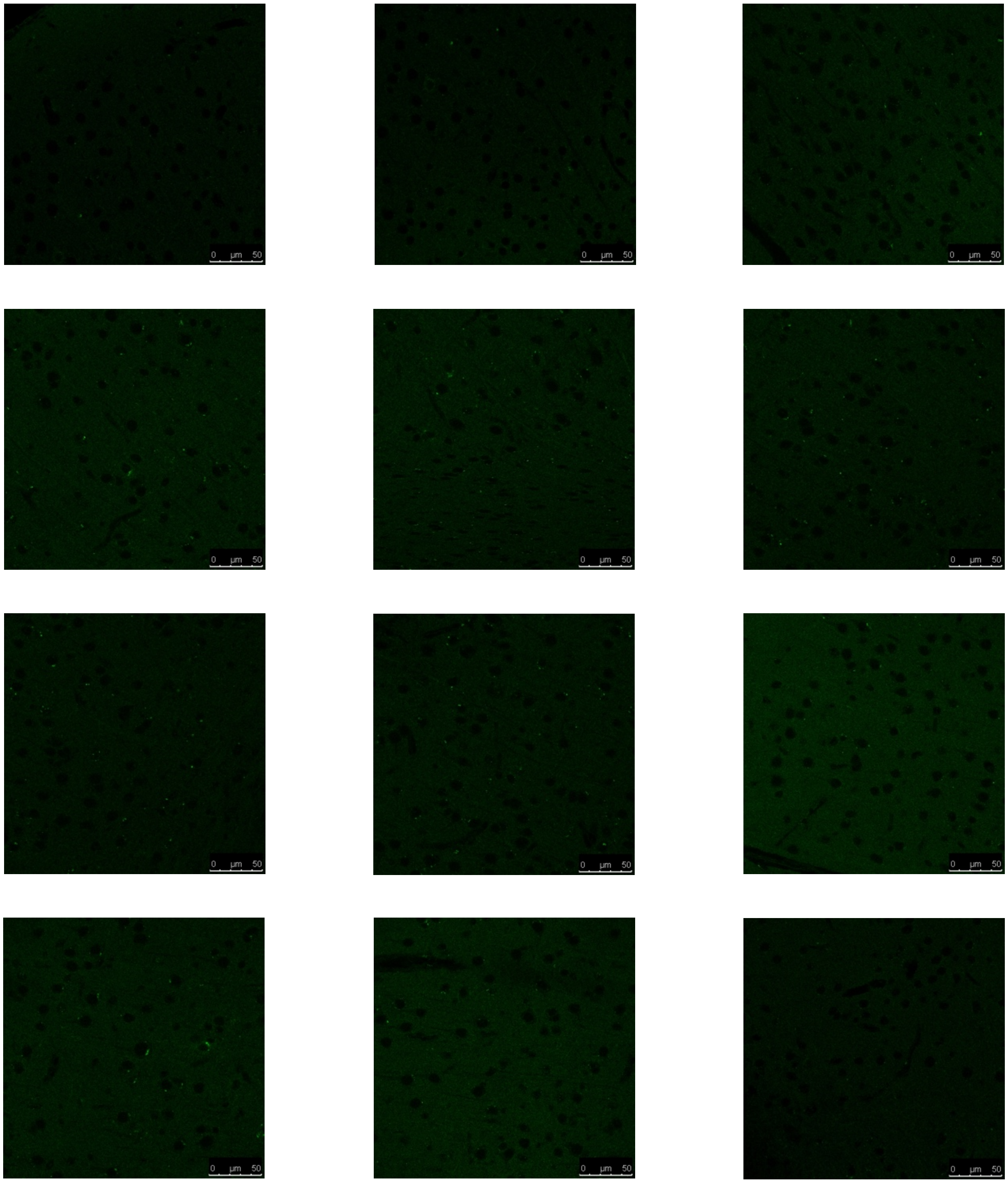

Raw images for Figure 2c (continued)

Cln3$^{\Delta ex7\text{-}8}$, UT

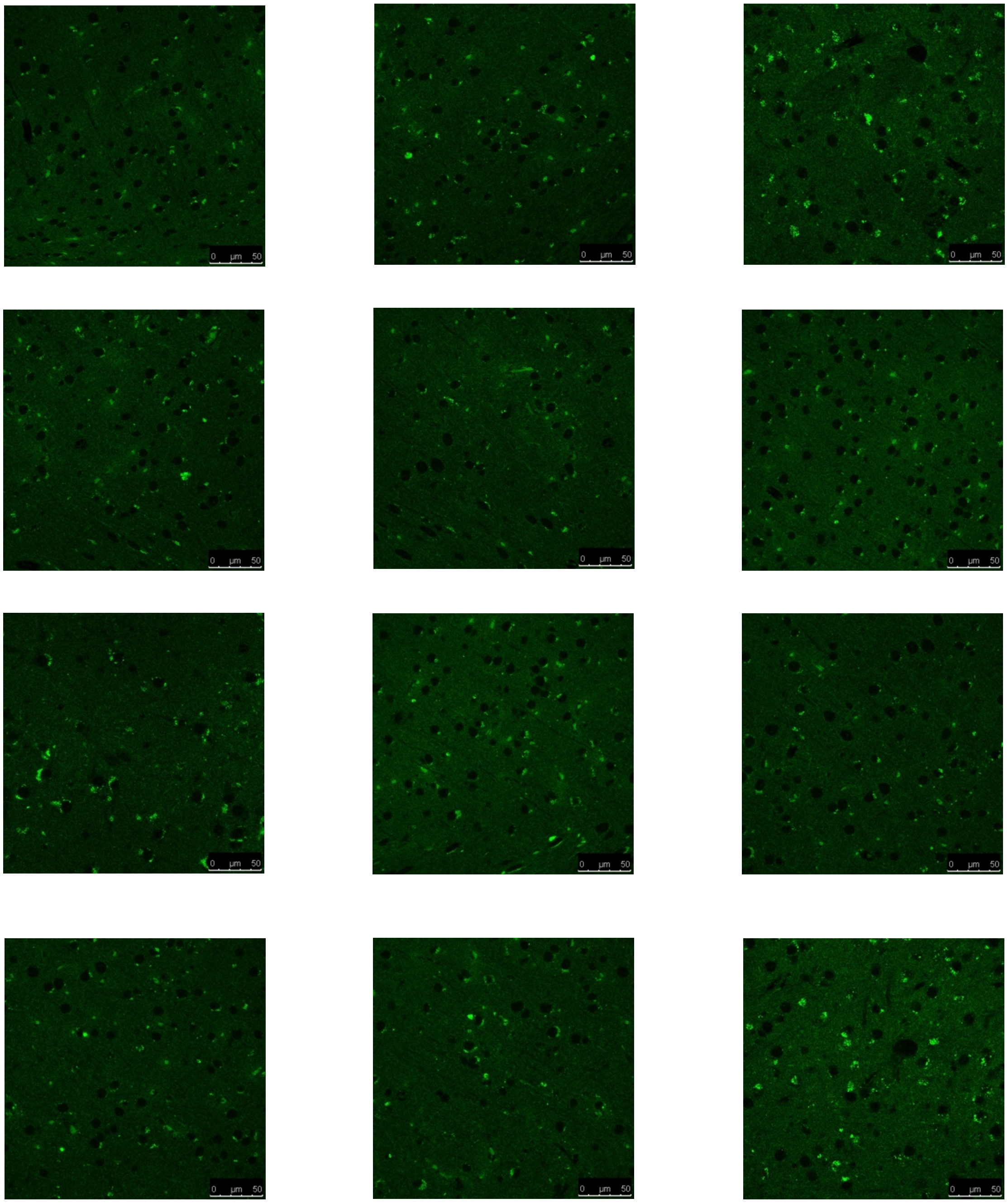

Raw images for Figure 2c (continued)

Cln3$^{\Delta ex7\text{-}8}$, Tre

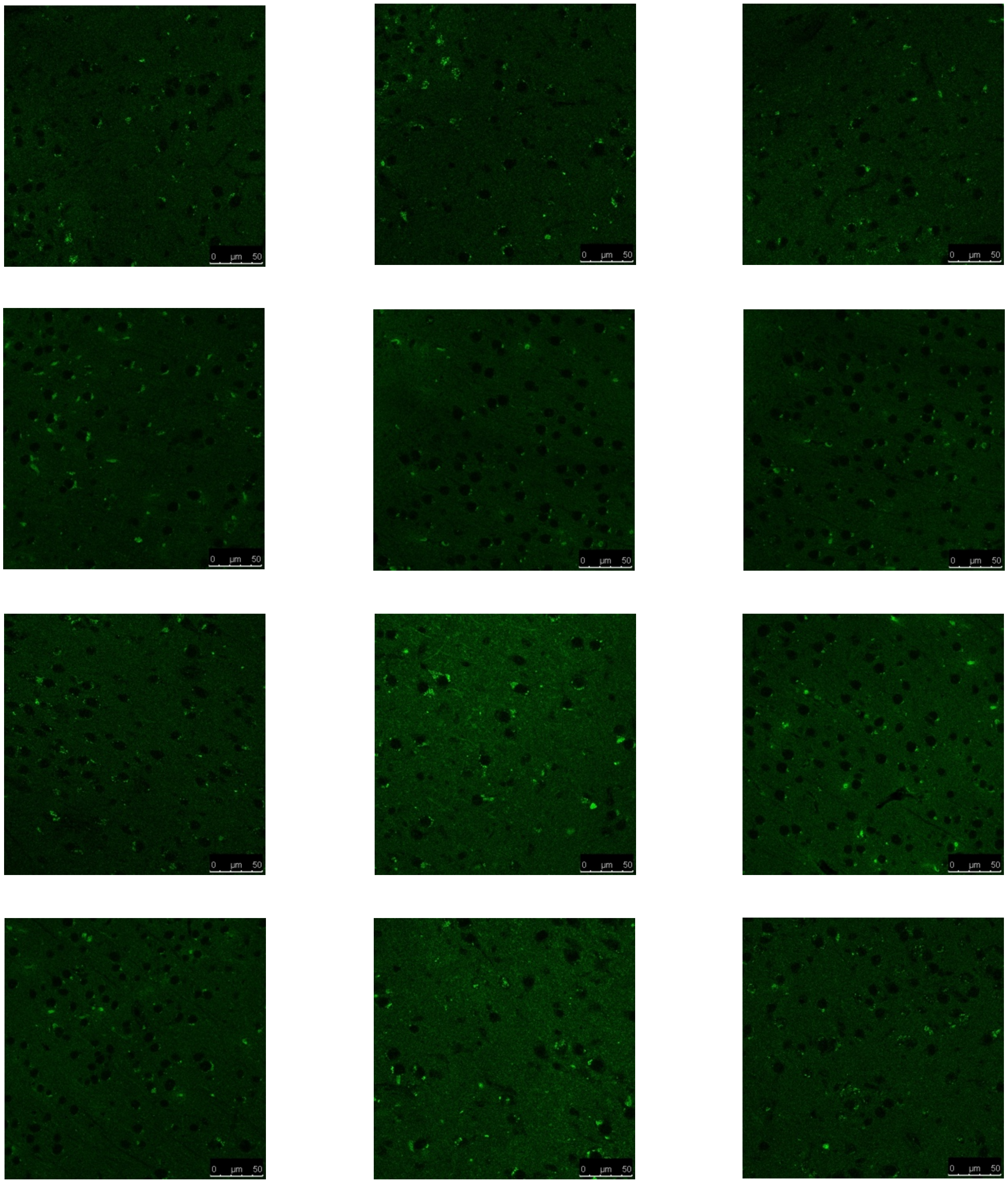

Raw images for Figure 3g

WT, UT

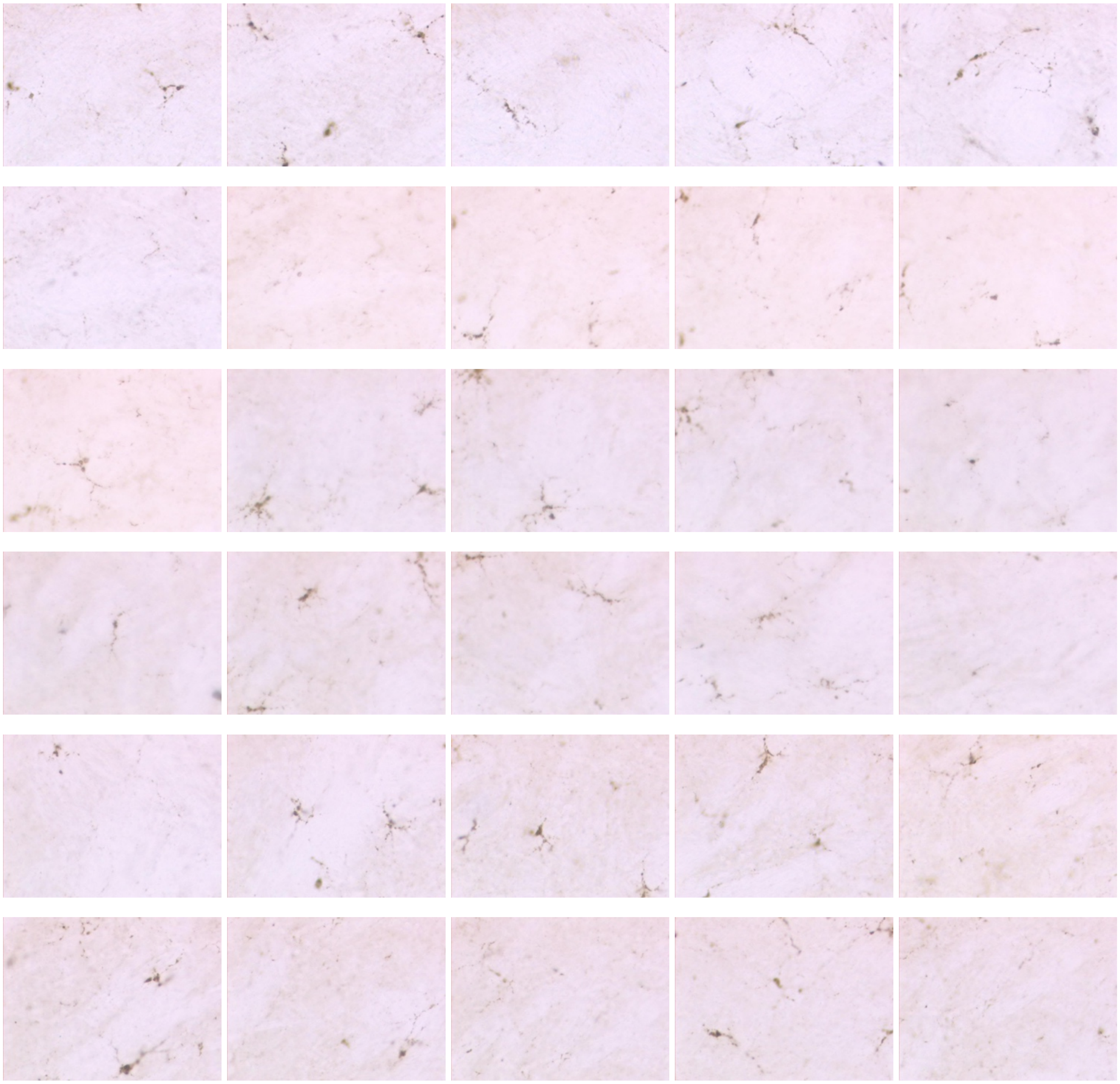

Raw images for Figure 3g (continued)

WT, UT

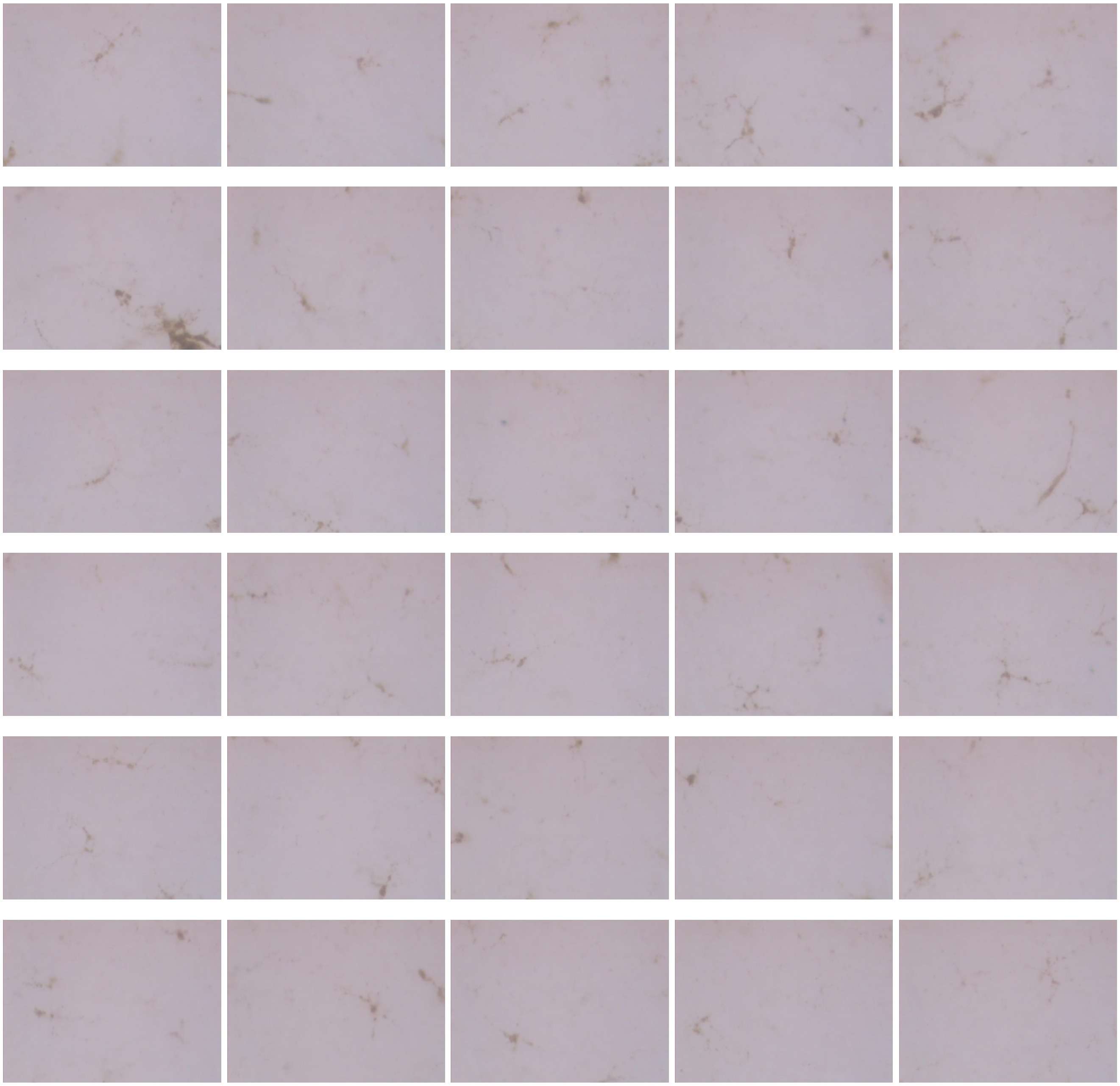

Raw images for Figure 3g (continued)

WT, UT

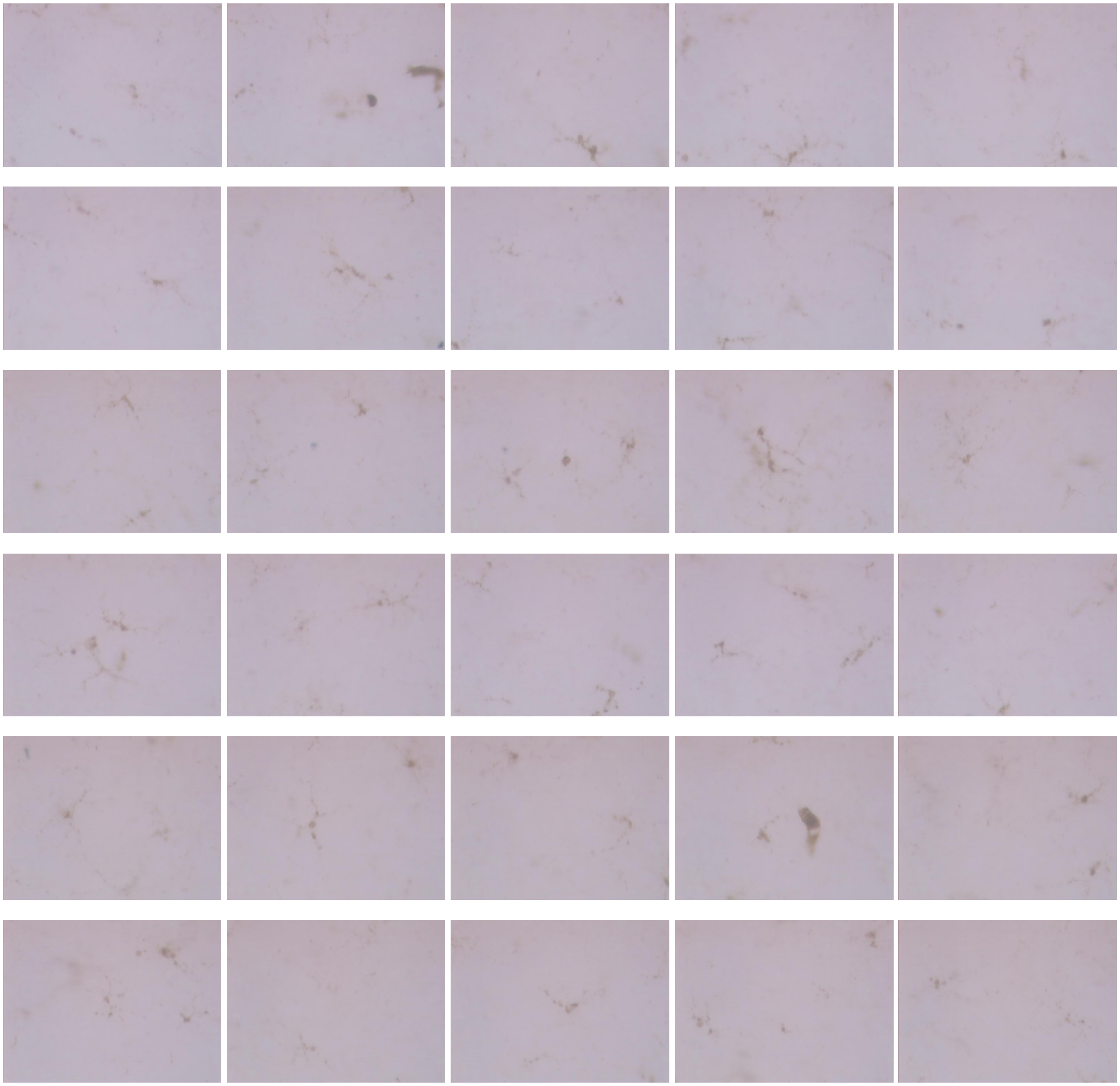

Raw images for Figure 3g (continued)

WT, UT

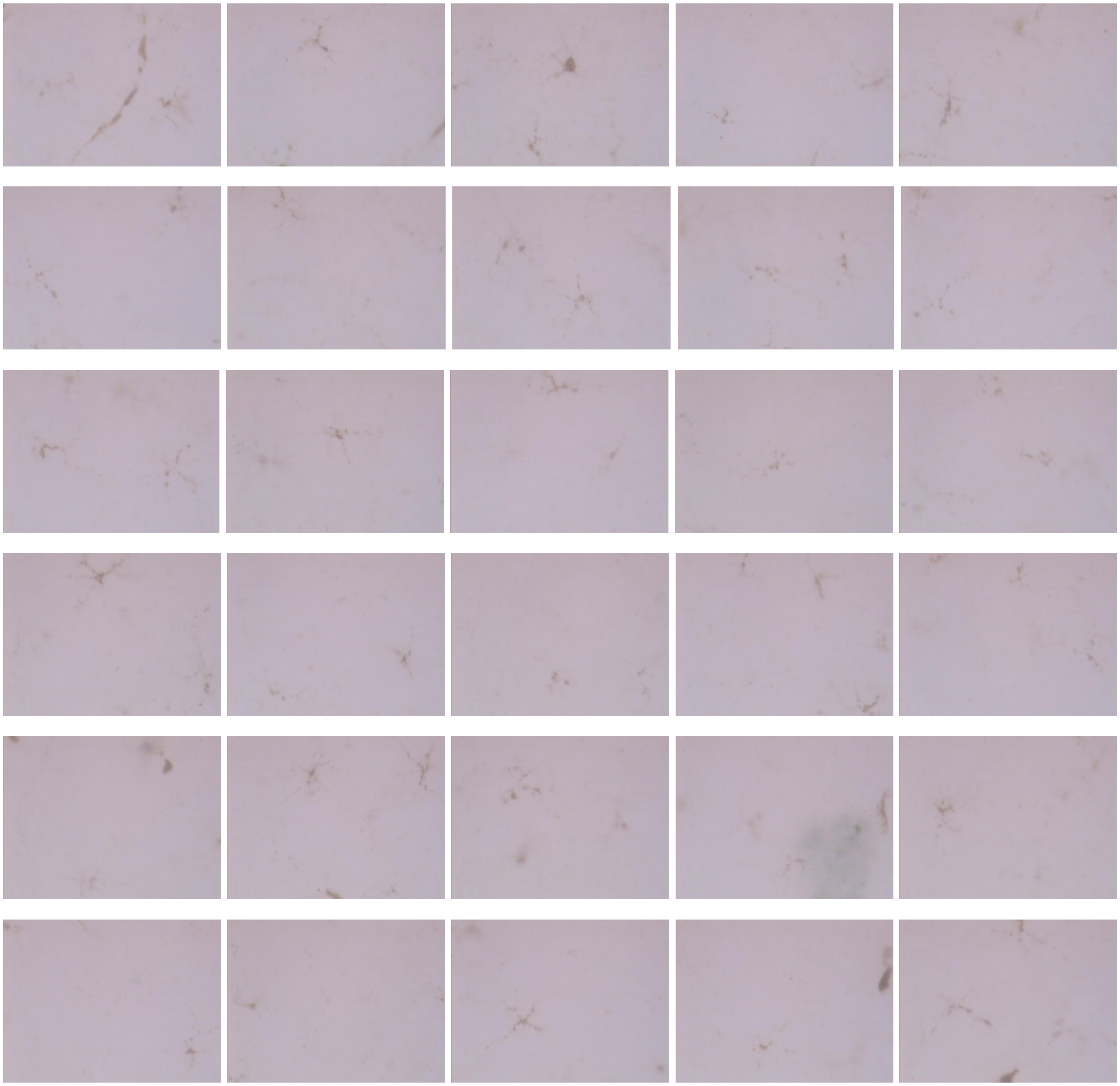

Raw images for Figure 3g (continued)

WT, Tre

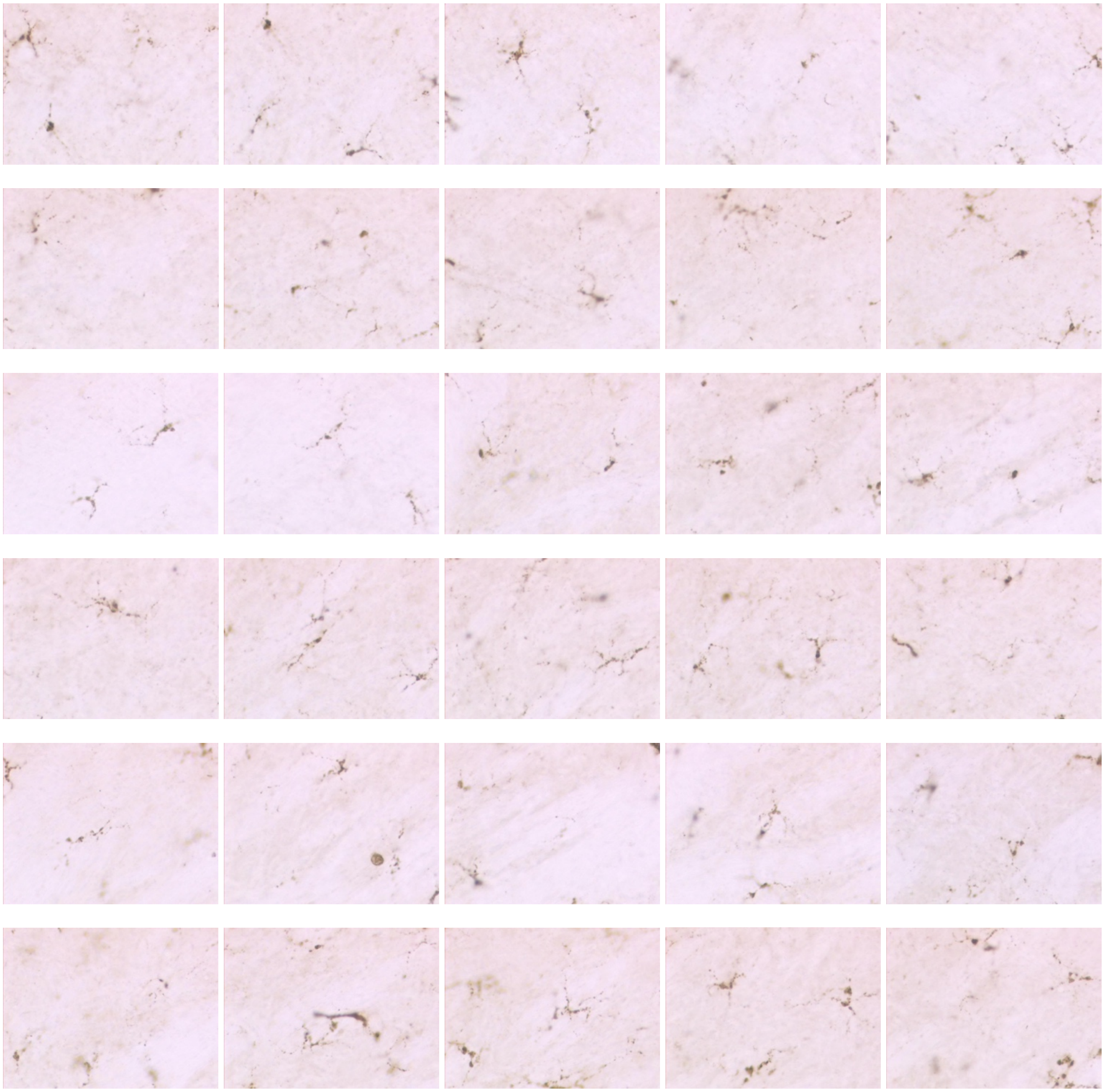

Raw images for Figure 3g (continued)

WT, Tre

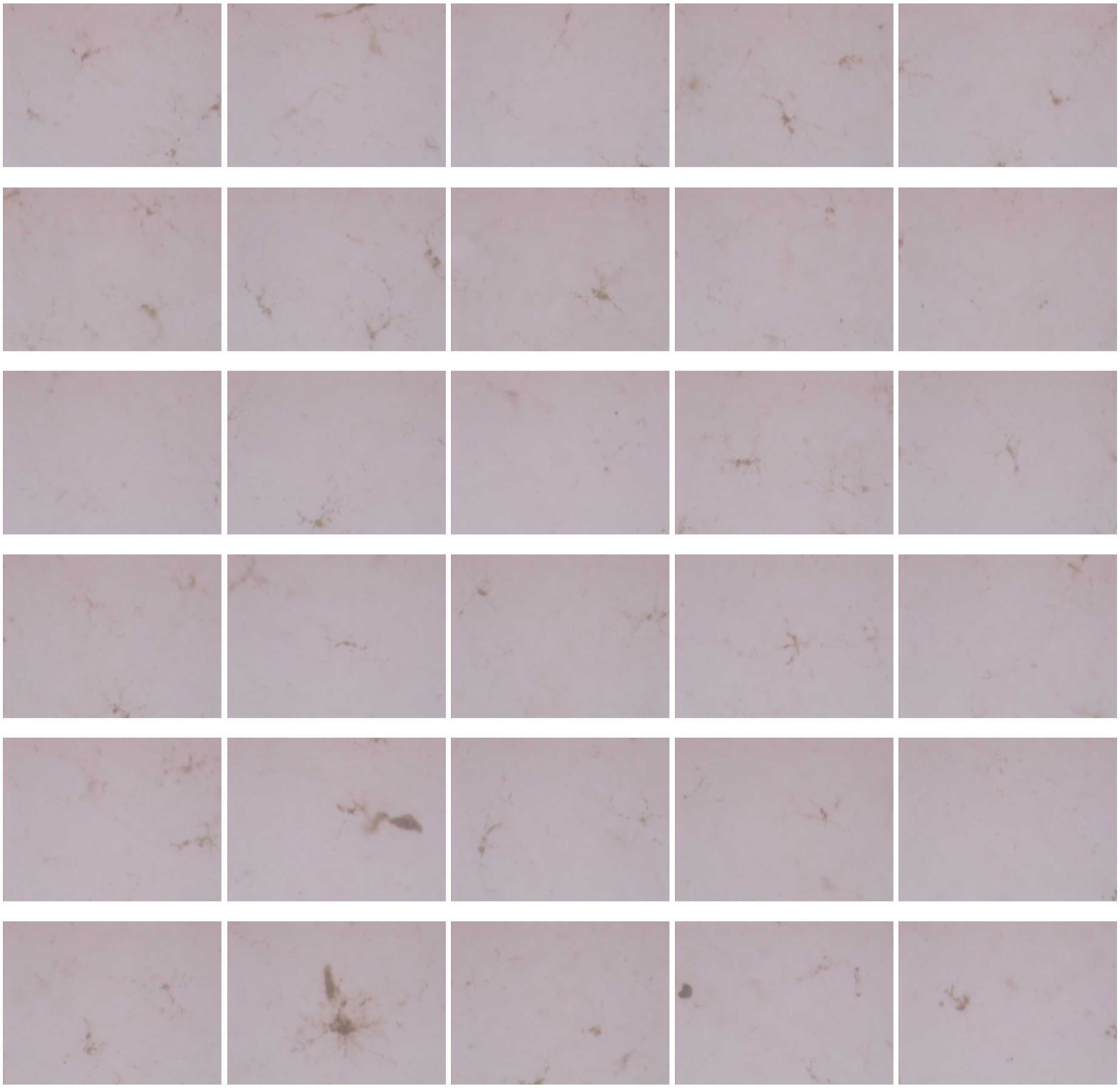

Raw images for Figure 3g (continued)

WT, Tre

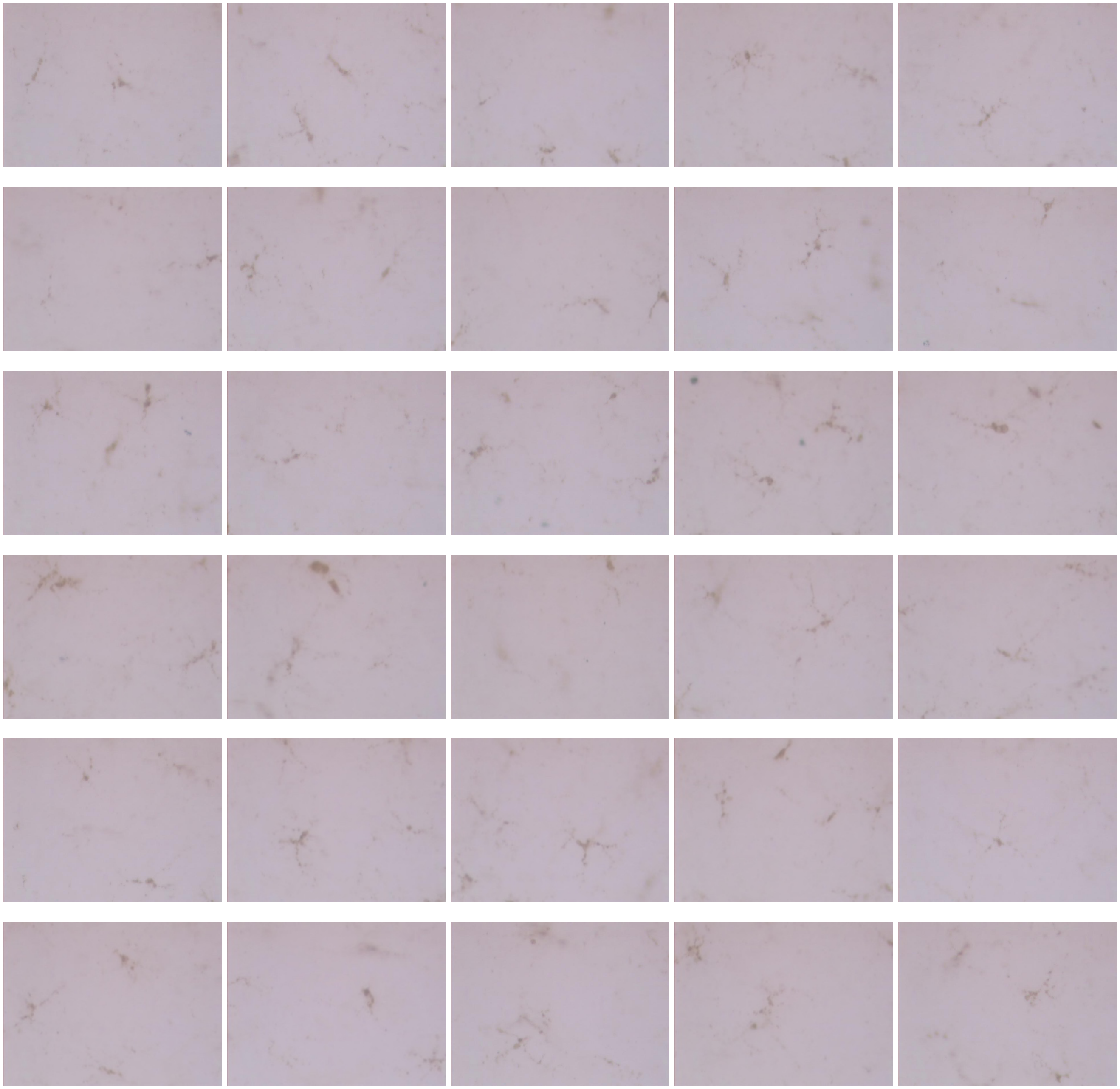

Raw images for Figure 3g (continued)

WT, Tre

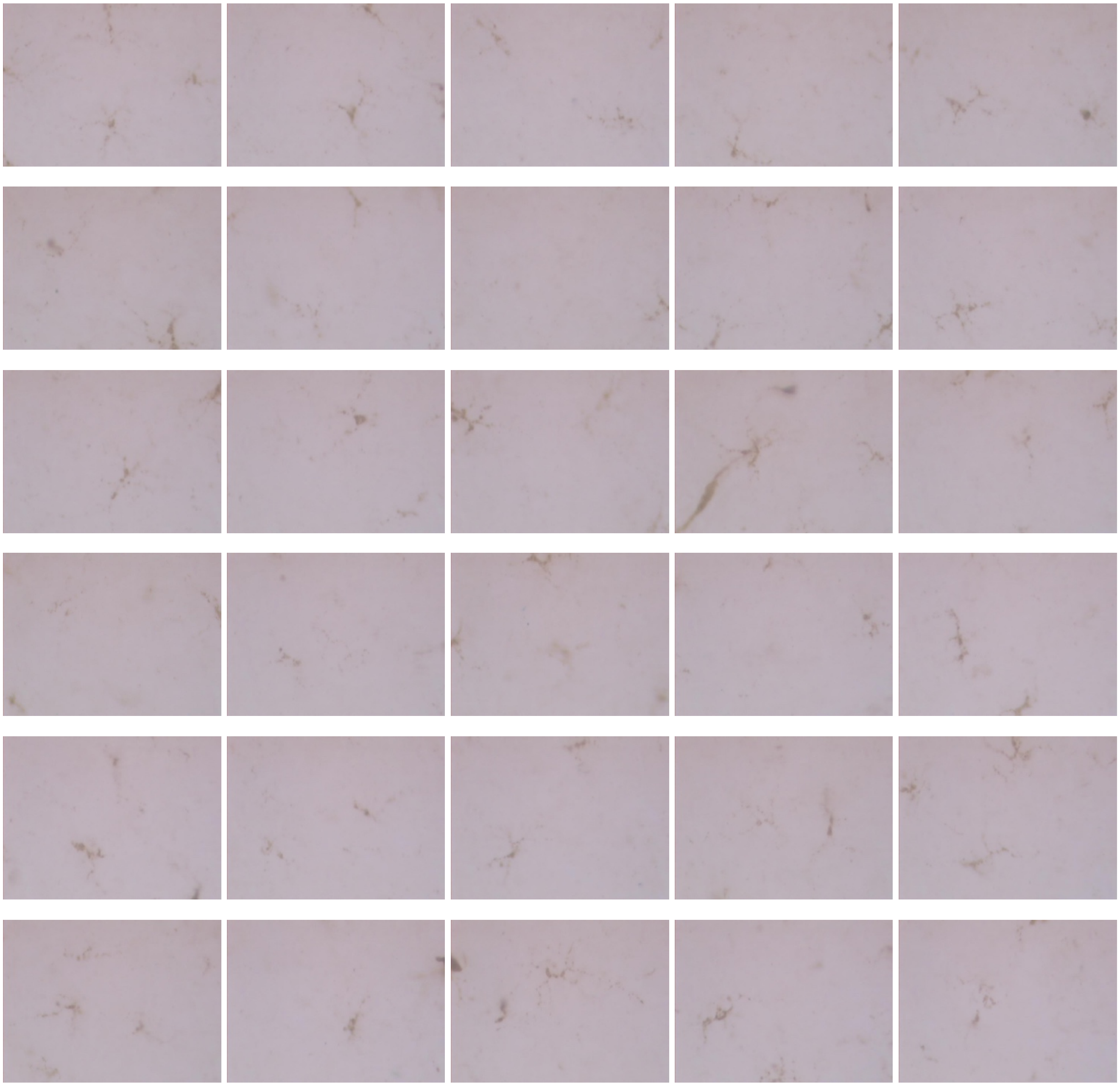

Raw images for Figure 3g (continued)

Cln3$^{\Delta ex7\text{-}8}$, UT

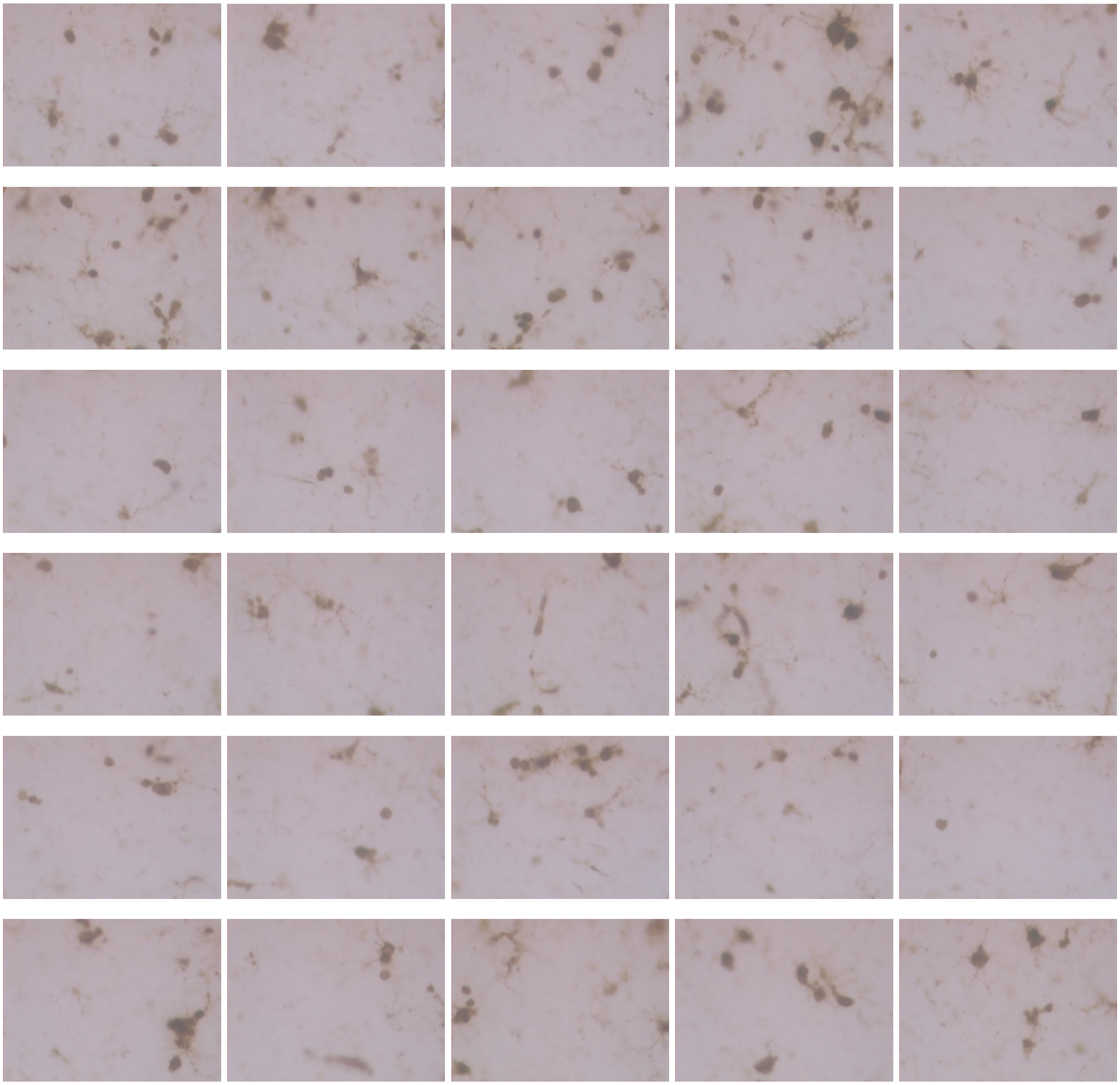

Raw images for Figure 3g (continued)

Cln3$^{\Delta ex7\text{-}8}$, UT

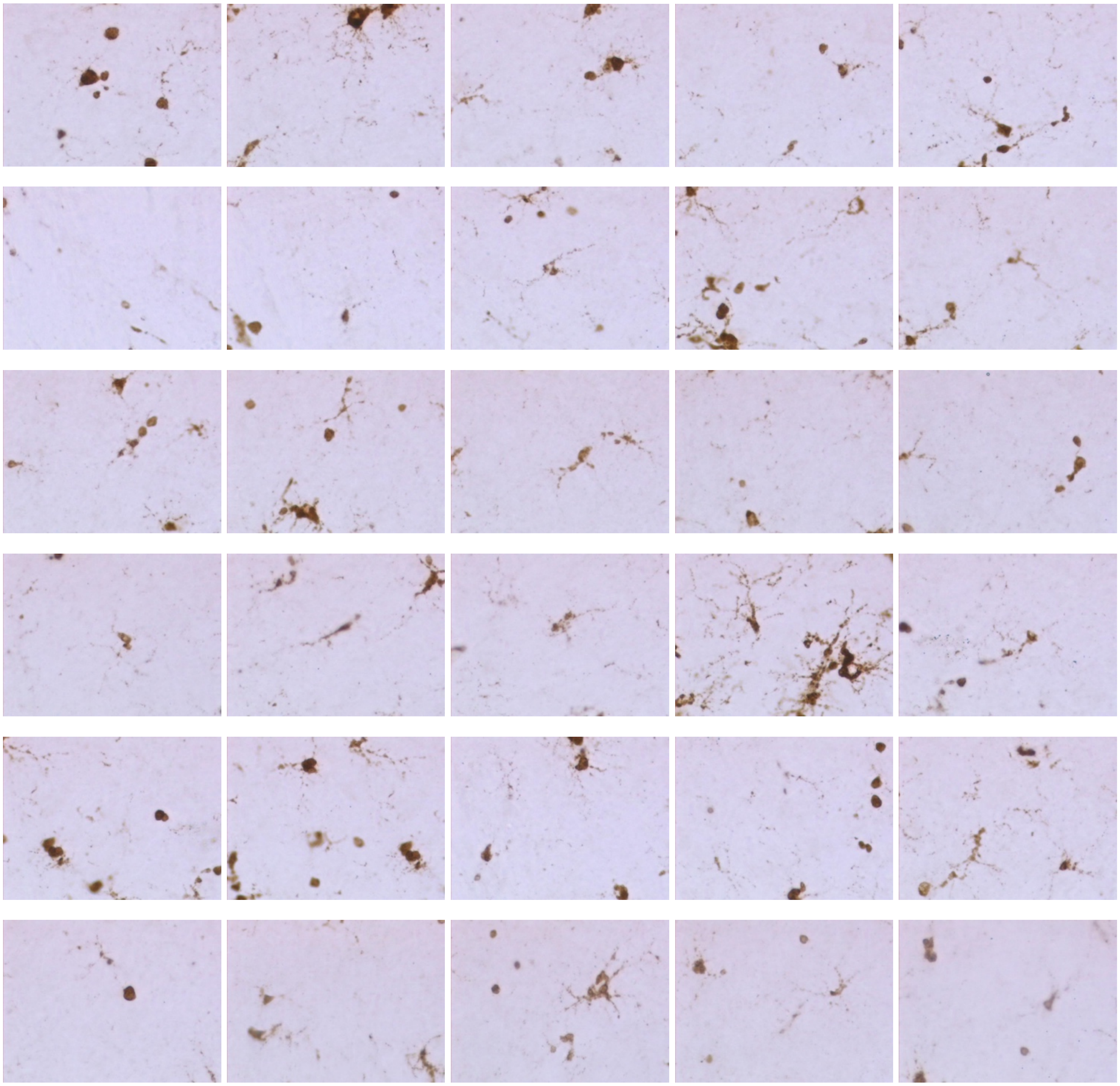

Raw images for Figure 3g (continued)

Cln3$^{\Delta ex7\text{-}8}$, UT

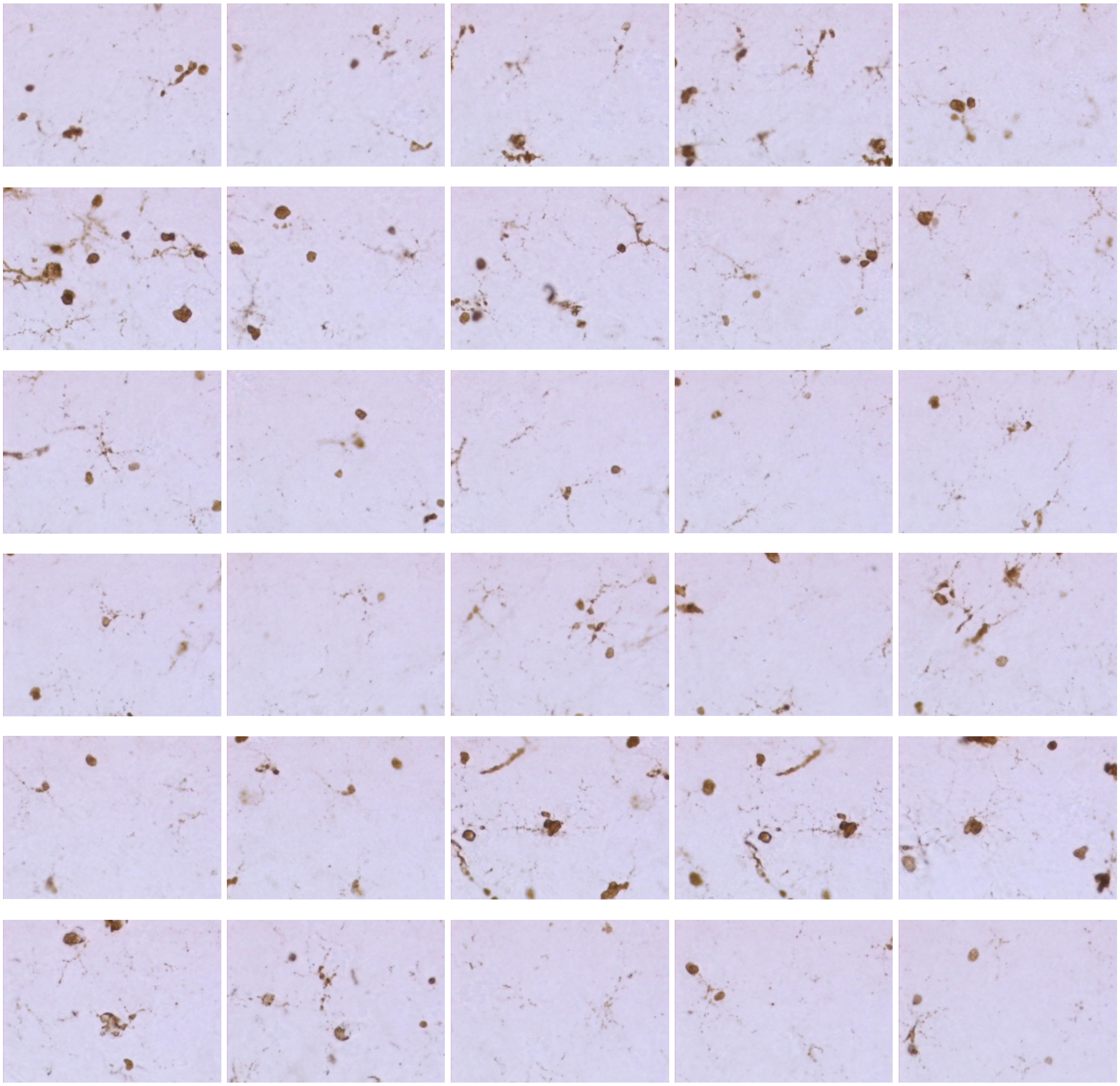

Raw images for Figure 3g (continued)

Cln3$^{\Delta ex7\text{-}8}$, UT

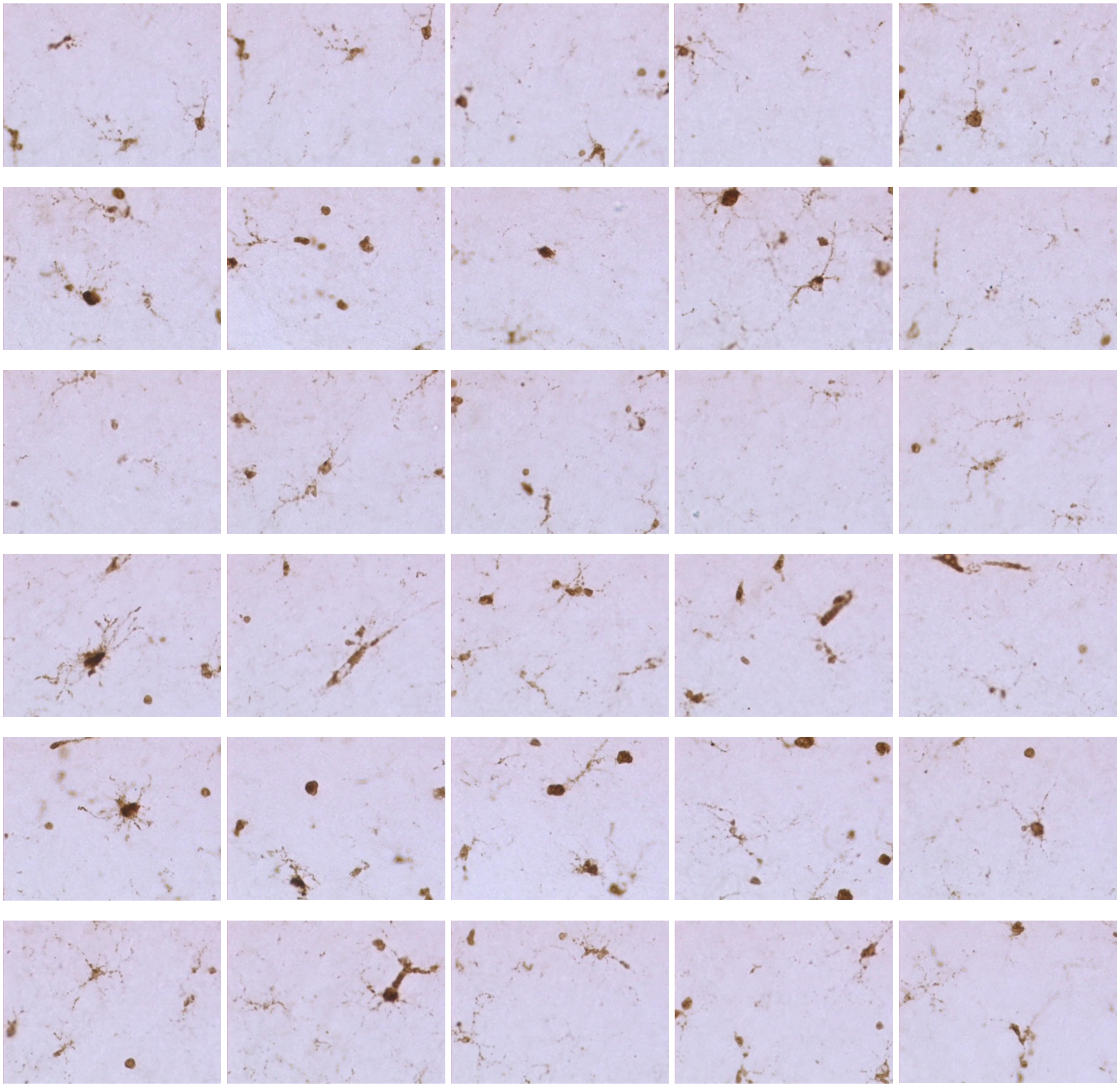

Raw images for Figure 3g (continued)

Cln3$^{\Delta ex7\text{-}8}$, Tre

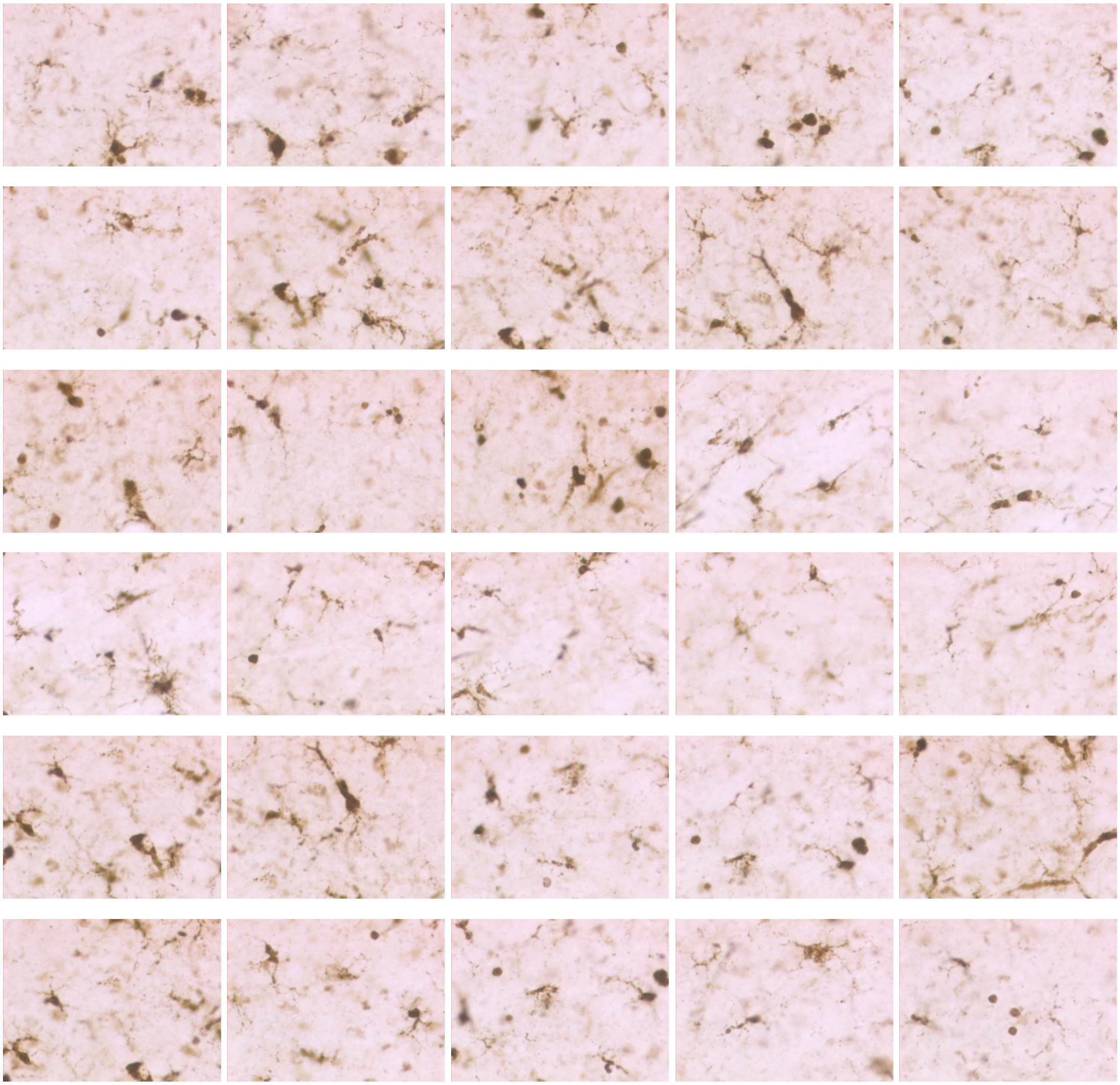

Raw images for Figure 3g (continued)

Cln3$^{\Delta ex7\text{-}8}$, Tre

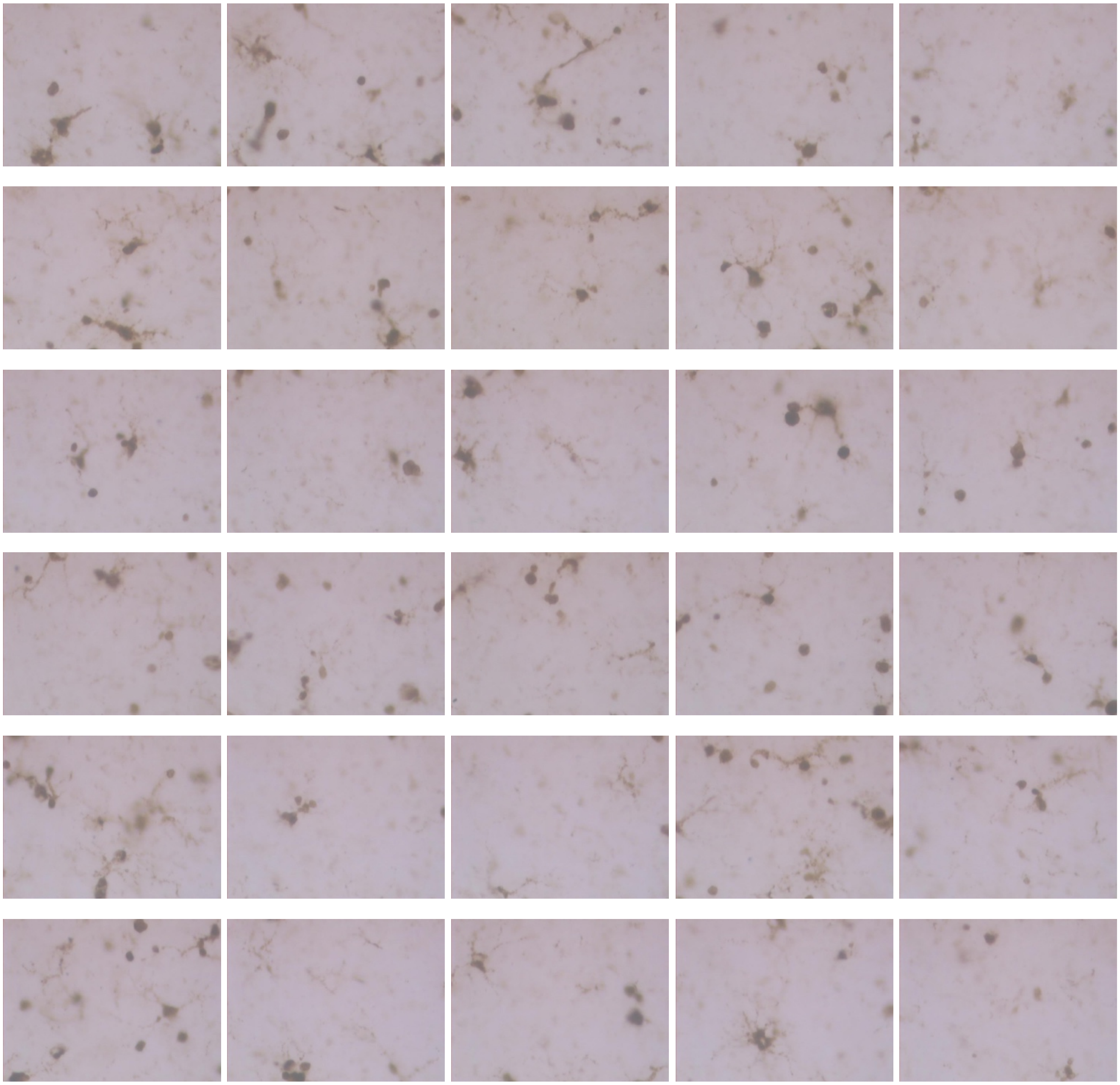

Raw images for Figure 3g (continued)

$Cln3^{\Delta ex7\text{-}8}$, Tre

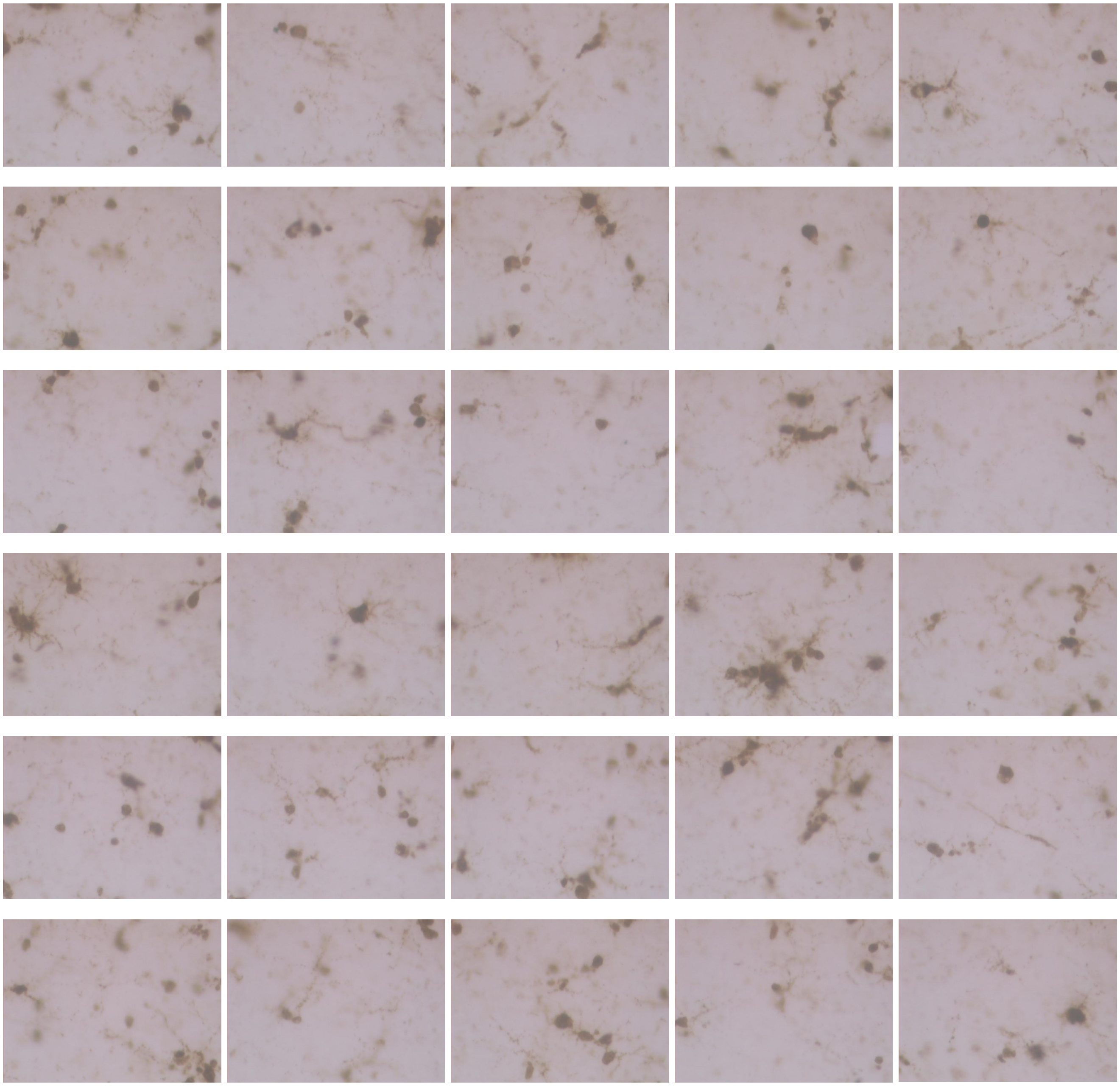

Raw images for Figure 3g (continued)

Cln3$^{\Delta ex7-8}$, Tre

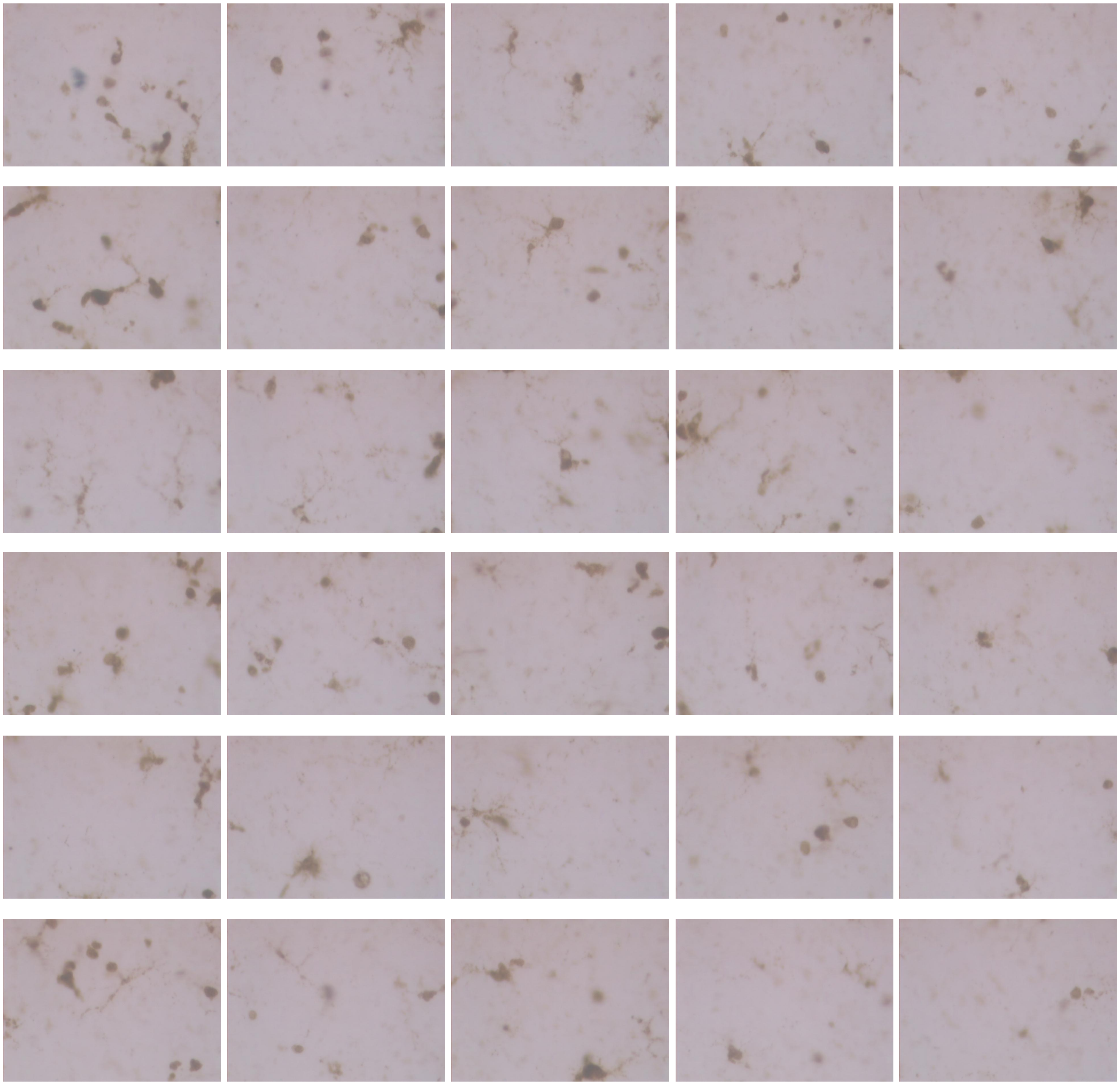

Supplement: Supplementary Information [file ncomms15793-s1.pdf]
